# A novel PKC activating molecule promotes neuroblast differentiation and delivery of newborn neurons in brain injuries

**DOI:** 10.1038/s41419-020-2453-9

**Published:** 2020-04-22

**Authors:** Samuel Domínguez-García, Noelia Geribaldi-Doldán, Ricardo Gómez-Oliva, Felix A. Ruiz, Livia Carrascal, Jorge Bolívar, Cristina Verástegui, Monica Garcia-Alloza, Antonio J. Macías-Sánchez, Rosario Hernández-Galán, Pedro Nunez-Abades, Carmen Castro

**Affiliations:** 1 Área de Fisiología, Facultad de Medicina, Universidad de Cádiz, Cádiz, Spain; 2 Instituto de Investigación e Innovación Biomédica de Cádiz (INiBICA), Cádiz, Spain; 3 Departamento de Anatomía y Embriología Humanas, Facultad de Medicina, Universidad de Cádiz, Cádiz, Spain; 4 Área de Nutrición, Facultad de Medicina Universidad de Cádiz, Cádiz, Spain; 5 Departamento de Fisiología, Facultad de Farmacia, Universidad de Sevilla, Sevilla, Spain; 6 Área de Bioquímica, Facultad de Ciencias, Universidad de Cádiz, Puerto Real, Spain; 7 Departamento de Química Orgánica, Facultad de Ciencias, Universidad de Cádiz, Puerto Real, Spain

**Keywords:** Target identification, Neural stem cells, Brain injuries

## Abstract

Neural stem cells are activated within neurogenic niches in response to brain injuries. This results in the production of neuroblasts, which unsuccessfully attempt to migrate toward the damaged tissue. Injuries constitute a gliogenic/non-neurogenic niche generated by the presence of anti-neurogenic signals, which impair neuronal differentiation and migration. Kinases of the protein kinase C (PKC) family mediate the release of growth factors that participate in different steps of the neurogenic process, particularly, novel PKC isozymes facilitate the release of the neurogenic growth factor neuregulin. We have demonstrated herein that a plant derived diterpene, (EOF2; CAS number 2230806-06-9), with the capacity to activate PKC facilitates the release of neuregulin 1, and promotes neuroblasts differentiation and survival in cultures of subventricular zone (SVZ) isolated cells in a novel PKC dependent manner. Local infusion of this compound in mechanical cortical injuries induces neuroblast enrichment within the perilesional area, and noninvasive intranasal administration of EOF2 promotes migration of neuroblasts from the SVZ towards the injury, allowing their survival and differentiation into mature neurons, being some of them cholinergic and GABAergic. Our results elucidate the mechanism of EOF2 promoting neurogenesis in injuries and highlight the role of novel PKC isozymes as targets in brain injury regeneration.

## Introduction

Brain injuries of different etiologies, produce sensory-motor alterations and irreversible cognitive deficits^1^. No effective treatments to compensate neuronal loss have been found so far. However, the discovery of neurogenesis in the adult brain revealing the capacity of the brain to generate new neurons from neural stem cells (NSC) has opened a door to new therapies to effectively treat this type of disorders. Adult neurogenesis occurs under physiological conditions in the dentate gyrus of the hippocampus (DG) and the subventricular zone (SVZ)^2,3^. These regions also react to an injury activating NSC to produce neural progenitor cells (NPC), stimulating proliferation and differentiation of NPC into neuroblasts, and altering migration patterns of these neuroblasts to lead them towards the injured area^4–6^. However, only a very small number of newly born neurons reach the lesion^7–9^. This is a consequence of the release of inflammatory signals that create a gliogenic/non-neurogenic environment within the injured tissue^10^. For example, ligands of the epidermal growth factor receptor (EGFR) in injuries promote gliogenesis and impair migration of neuroblasts towards injuries^11^. On the contrary, inhibition of EGFR ligand release facilitates neurogenesis in injuries^12–14^. Transforming growth factor alpha (TGFα), and other EGFR ligands are synthesized as precursor membrane anchored proteins, which are secreted upon the metalloprotease-catalyzed proteolysis of the soluble, active ectodomain. ADAM17 is the main convertase involved in the ectodomain shedding of TGFα and other EGFR ligands^15–17^. However, ADAM17 also catalyzes the proteolysis of ligands, that activate other receptors of the ErbB family such as neuregulins. The selectivity of this enzyme for each ligand governs ligand release and it is determined by phosphorylation reactions within the cytoplasmic domain of the pro-ligand molecules, catalyzed by kinases of the protein kinase C (PKC) family^18^. PKCα activated by phorbol-12-myristate-13-acetate (PMA) catalyzes the phosphorylation of TGFα, amphiregulin and HB-EGF precursors facilitating their ADAM17-mediated shedding. On the contrary, activation of novel PKCδ is required for ADAM17-mediated secretion of neuregulin 1 (NRG1)^18,19^. Neuregulin-mediated activation of ErbB4 favors neurogenesis in the adult brain promoting neuroblast survival and organizing migration of neuroblasts from the SVZ towards the olfactory bulb^20,21^. Considering these findings, it seems reasonable to hypothesize that specific activation of different PKC isozymes would alter the pattern of growth factor secretion within neurogenic niches, and will regulate either proliferation—mediated by classical PKCβ and α^22^—or neuroblast survival and migration—mediated by novel PKC isozymes^23^. Consequently, in order to promote neurogenesis in injuries, it would be useful to find molecules with the capacity to activate novel PKC specifically facilitating neuregulins release. Such molecules would probably promote differentiation of NPC into newborn neurons and enable neuroblasts migration from neurogenic areas towards injuries.

## Results

### The diterpene with lathyrane structure EOF2 activates PKC without promoting proliferation

The lathyrane EOF2 (CAS number 2230806-06-9) was previously described by our group as a diterpene with no effect on NPC proliferation^22^. Since other diterpenes induced proliferation of NPC^24^ via classical PKC activation^23^, we analyzed its capacity to activate PKC and to facilitate the generation of neurospheres. Although EOF2 treatment increased PKC activity by twofold in neurosphere cultures (supplementary fig. S1 D), it exerted no change in the size of the neurospheres and a reduction in the number of neurospheres generated. Given its capacity to activate PKC without inducing proliferation, it was possible that EOF2 specifically activated novel PKC without affecting the classical isozymes—reported as responsible of the proliferative effect^22^.

### EOF2 facilitates NRG1 release

**Fig. 1:**
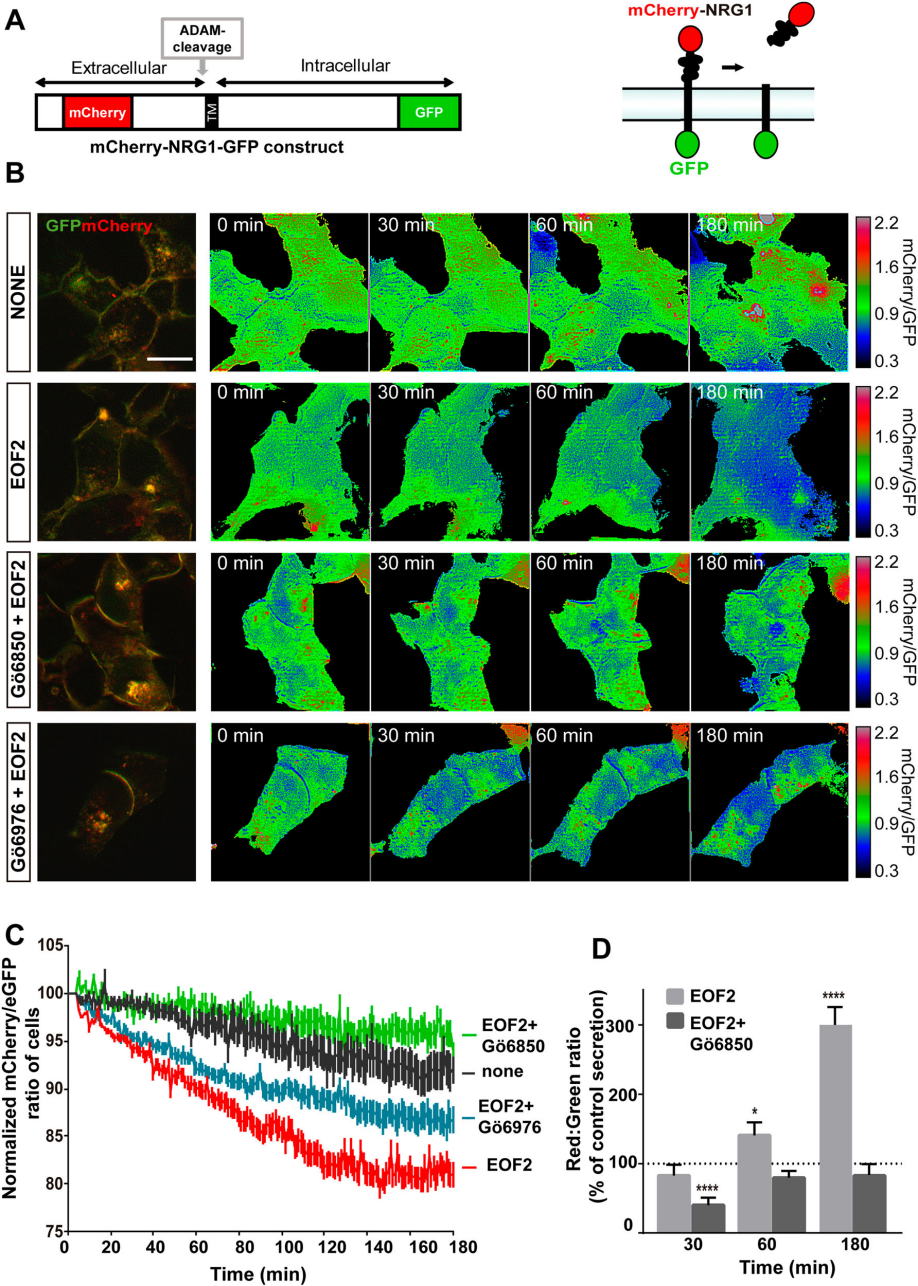
Real-time imaging of the EOF2 induced ectodomain shedding of Cherry-NRG1-GFP. **a** Schematic representation of the mCherry-NRG1-GFP construct and mechanism of action. **b** Images of mCherry-NRG1-GFP expressing, serum-starved HEK293T cells stimulated with diluent, EOF2 (5 µM), EOF2 plus the general PKC inhibitor Gö6850 (5 µM) and EOF2 plus the classical inhibitor of PKC Gö6976 (1 µM) during 180 min. PKC inhibitors were added 30 min before EOF2. mCherry/GFP ratio images at the indicated time points are shown in the intensity modulated display-mode. The color range goes from red to blue to represent mCherry/GFP ratios. The upper and lower limits of the ratio range are shown on the right. Scale bar represent 20 µm. **c** Quantitative analysis of the microscopic images obtained from the time-lapse assays of HEK293T cells expressing Cherry-NRG1-GFP. mCherry/GFP ratios were normalized to the average mCherry/GFP ratio measured before stimulation. The mean normalized mCherry/GFP ratios and SEM are shown, *n* = 40. See also supplementary movie 1. **d** Fluorescence analysis of m-Cherry in the culture medium of HEK293T cells transfected with the construct mCherry-NRG1-GFP as a ratio of GFP fluorescence in the cellular fraction. mCherry signal in the culture medium of transfected HEK293T cells was measured in a fluorimeter as described in the Material and Methods section. Bars represent the ratio red:green in transfected cells at different time points after treatment with EOF2 or EOF2 + PKC inhibitor Gö6850. Values are given as a percentage of the red:green ratio in non-treated transfected cells (dashed line). Data are the mean values ± S.E.M of nine independent measurements (*n* = 9). Statistical analysis: two tailed unpaired Student’s *t* test (*EOF2 vs control at 60 min, *p* = 0.0456; ****EOF2 + Gö6850 vs control at 30 min, *p* < 0.0001; EOF2 vs control at 180 min, *p* < 0.0001).

**Fig. 2:**
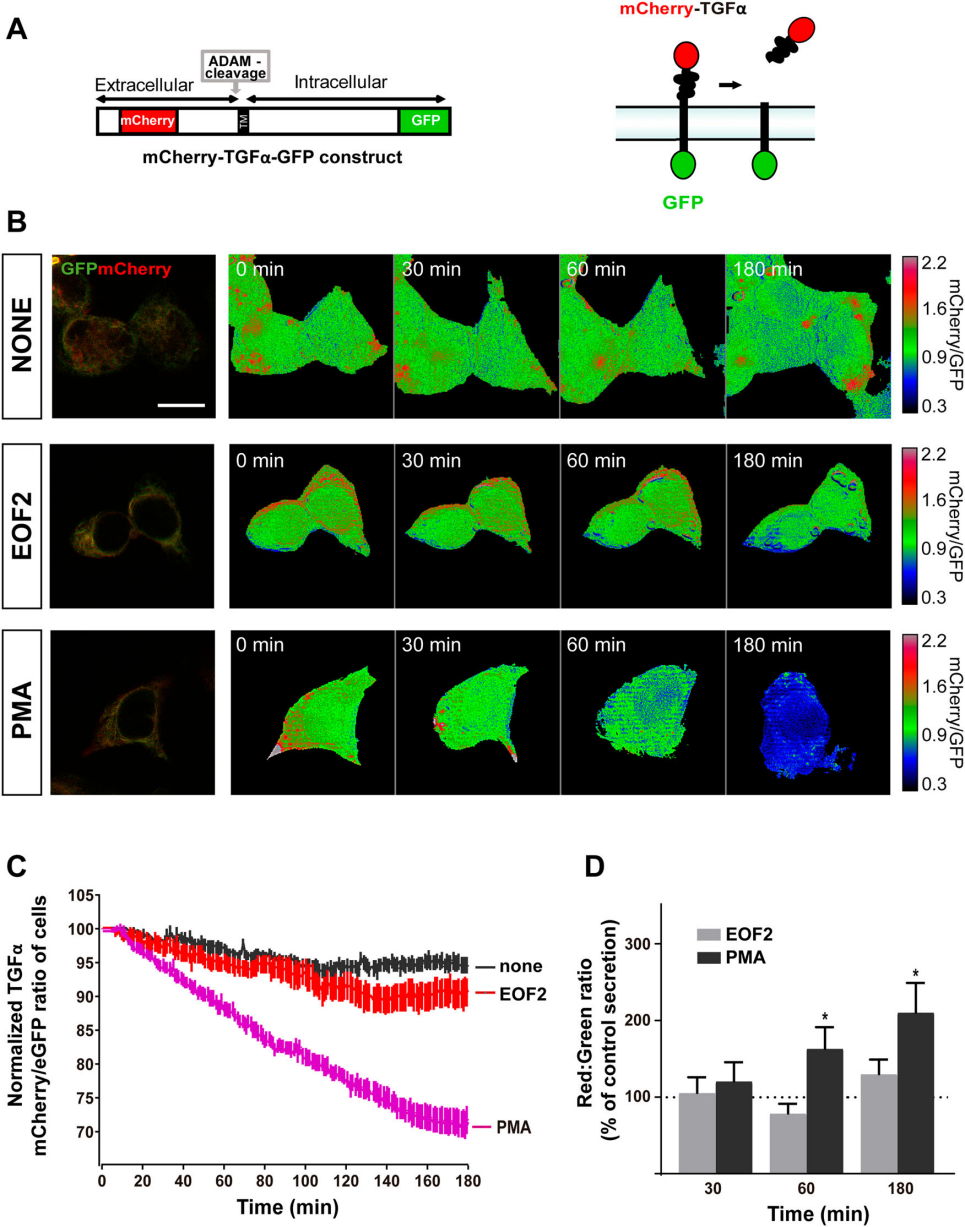
Real-time imaging of the EOF2 induced ectodomain shedding of Cherry-TGFα-GFP. **a** Schematic representation of the mCherry-TGFα-GFP construct and mechanism of action. **b** Images of mCherry-TGFα-GFP expressing, serum-starved HEK293T cells stimulated with diluent, EOF2 (5 µM) or PMA (5 µM) during 180. mCherry/GFP ratio images at the indicates time points are shown in the intensity modulated display-mode. The colors range goes from red to blue to represent mCherry/GFP ratios. The upper and lower limits of the ratio range are shown on the right. Scale bar represent 20 µm. **c** Quantitative analysis of mCherry/GFP ratios in HEK293T cells expressing Cherry-TGFα-GFP. mCherry/GFP ratios were normalized to the average mCherry/GFP ratio measured before stimulation. The mean normalized mCherry/GFP ratios and SEM are shown, *n* = 40. See also supplementary movie 2. **d** Fluorescence analysis of m-Cherry in the culture medium of cells transfected with the fusion protein construct mCherry-TGFα-GFP as a ratio of GFP fluorescence in the cellular fraction. mCherry signal in the culture medium of transfected HEK293T cells was measured in a fluorimeter as described in the Material and Methods section. Bars represent the ratio red:green in transfected cells at different time points after treatment with EOF2 or EOF2 + PKC inhibitor Gö6850 as a percentage of the red:green ratio in non-treated transfected cells (dashed line). Cells were treated with the EOF2 at the indicated times. Data are the mean values ± S.E.M of nine independent measurements (*n* = 9). Statistical analysis: two tailed unpaired Student’s *t* test: (*PMA vs control at 60 min *p* = 0.0403; *PMA vs control at 180 min *p* = 0.0112).

In order to determine whether EOF2 specifically induced neuregulin release, we used protein constructs in which NRG1 was fused to mCherry in the N-terminal and eGFP in the C-terminal (mCherry-NRG1-eGFP) (Fig. 1A) according to the work of Kamezaki et al. with modifications^25^. A similar construct was used to test TGFα release (mCherry-TGFα-eGFP) (Fig. 2A). We tested the effect of EOF2 in HEK293T cells transfected with the constructs. As shown in supplementary table T1, HEK293T cells express the mRNAs encoding for PKC isozymes involved in neuregulins and TGFα release as well as ADAM17 protein, validating them as a model to test the release of the above-mentioned growth factors.

NRG1 release was measured as explained in M&M section. As shown in Fig. 1 the red/green ratio in HEK293T transfected with mCherry-NRG1-eGFP and treated with vehicle (none) did not vary with time (Fig. 1B, C) (supplementary movie 1), indicating that in the absence of a PKC-stimulating compound this ligand was not released. However, the addition of EOF2 induced a progressive reduction of this ratio (Fig. 1B, C) (supplementary movie 2), suggesting that this compound was facilitating the mCherry-labeled ectodomain shedding of neuregulin. Interestingly, these changes in color ratio were prevented by addition of the general PKC inhibitor Gö6850 to the culture medium (at a final concentration of 1 µM), 30 min before the beginning of the treatment with EOF2 (Fig. 1B, C) (supplementary movie 3). In order to uncertain the role that novel PKC played in this effect, we added the classical PKC inhibitor Gö6976 (at a final concentration of 1 µM), 30 min before the addition of EOF2 to mainly inhibit classical PKC. Results show a reduction in the red/green ratio even in the presence of the inhibitor, indicating that the effect on neuregulin release was mediated by novel PKC activation (Fig. 1A–C) (supplementary movie 4).

Furthermore, we analyzed the levels of mCherry signal in the culture medium of HEK293T cells transfected with the mCherry-NRG1-eGFP construct and treated with EOF2, vehicle, or EOF2 + Gö6850. 30, 60, and 180 min after the treatment cells were separated from the culture medium and mCherry fluorescence was measured in a fraction of 50 µL of medium (total volume of medium 1 mL) as a ratio of the GFP fluorescence remaining inside the cells. The red/green ratio in control cultures (non-treated transfected cells) at all time points was considered as 100% (Fig. 1D dashed line). A time-dependent increase in the red/green fluorescence ratio was observed in the culture medium upon treatment with EOF2. This was not observed in cultures treated with EOF2 + Gö6850 (Fig. 1D).

In order to analyze whether EOF2 specifically facilitated the shedding of NRG1, and had no effect on TGFα, we used the construct mCherry-TGFα-eGFP (Fig. 2A) transfected into HEK293 treated with EOF2. (Fig. 2B, C). Time-lapse imaging experiments showed no reduction in the red/green ratio upon treatment with either vehicle or EOF2 (supplementary movies 5–6), whereas a time dependent reduction was observed with the pan-PKC activator PMA (supplementary movie 7). Likewise, no alteration of the red/green ratio was observed when mCherry was measured in the culture medium of mCherry-TGFα-eGFP transfected cells at different time points (Fig. 2D).

### EOF2 facilitates neuronal differentiation of SVZ isolated progenitors in vitro

**Fig. 3:**
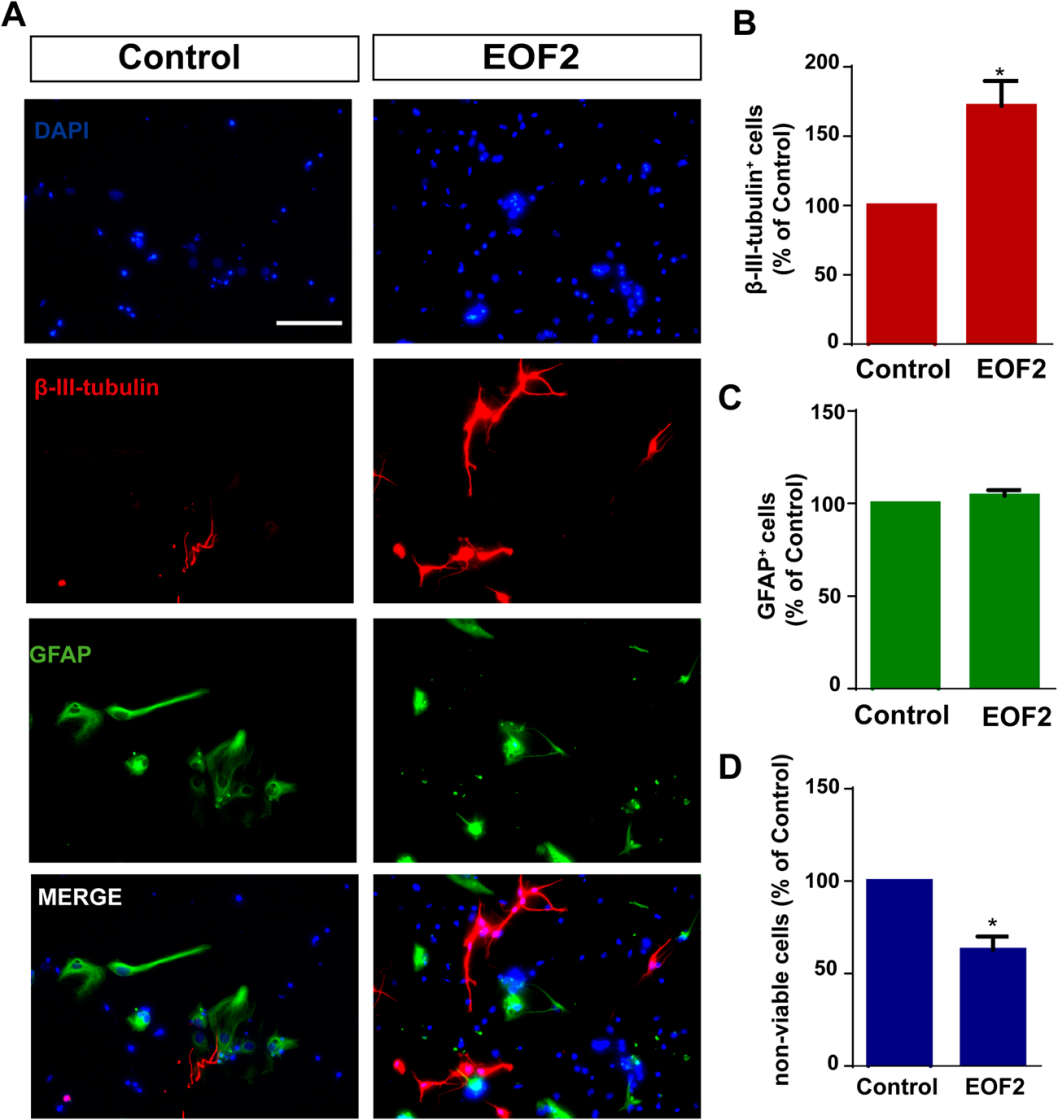
EOF treatment promotes differentiation of NPC to neurons. **a** Representative fluorescence microphotographs of SVZ-derived cultured NPC that had been treated with either diluent (control) or EOF2 (5 µM). Cells were grown without growth factors and allowed to differentiate for 72 h after treatment and then fixed. β-III-tubulin marker was used for neuronal immunodetection (red) and glial cells were identified by the immunodetection of GFAP (green). Total nuclei were counterstained with DAPI (blue). Scale bar = 50 µm. **b** Graph represents the percentage of total cells (detected by DAPI nuclear staining) that were positive for β-III-tubulin expression expressed as percentage of control. Data are the means ± S.E.M.; *n* = 9 independent values (*n* = 9). Statistical analysis: *p = 0.0025 in unpaired two tailed Student’s *t* test comparing with the control group. **c** Graph represents the percentage of total cells (detected by DAPI nuclear staining) that were positive for GFAP expression expressed as percentage of control. Data are the means ± S.E.M. of nine independent values (*n* = 9). **d** Graph represents the percentage of nonviable cells after treatment as a percentage of total cells (detected by DAPI nuclear staining) expressed as a percentage of control. Viability was measured by trypan blue exclusion as described in the methods section. Results show a statistically significant increase in the percentage of β-III-tubulin^+^ cells whereas no change of GFAP^+^ cells in the presence of EOF2. Data are the means ± S.E.M. of nine independent values (*n* = 9). Statistical analysis: *p = 0.0004 in unpaired two tailed Student’s *t* test comparing with the control group.

To elucidate whether EOF2 played a role in neurogenesis, we tested the capacity of this compound to induce neuroblast enrichment in cultures of SVZ-isolated cells under differentiation conditions. Cells were cultured attached onto a substrate in the absence of growth factors and left for 72 h to differentiate in the presence and absence of EOF2. The percentage of neuroblasts (β-III-tubulin^+^) and glial cells (GFAP^+^) was quantified. In control cultures the percentage of GFAP^+^ cells and β-III-tubulin^+^ cells were similar (Fig. 3A–D). Treatment of cultures with EOF2 increased the percentage of β-III-tubulin^+^ cells in almost twofold (Fig. 3A–B), whereas the percentage of GFAP^+^ cells remained unchanged (Fig. 3A–C). In addition, a small reduction in the number of nonviable cells in cultures treated with EOF2 was observed (Fig. 3D), suggesting an additional role for EOF2 in promoting survival.

### EOF2-induced differentiation of SVZ isolated progenitors in vitro is mediated by novel PKC

**Fig. 4:**
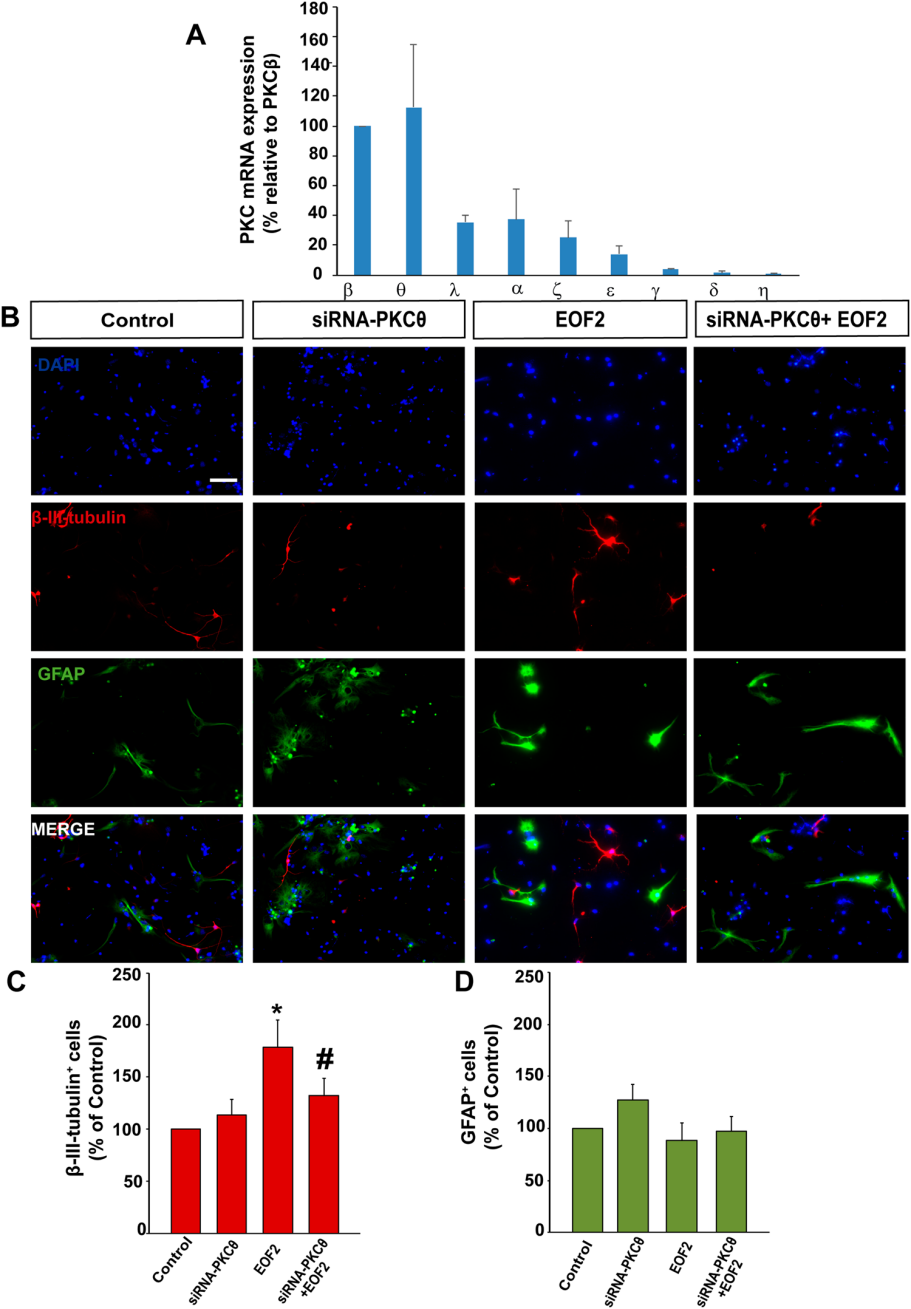
EOF2 induce neuronal differentiation via PKCθ activation without affecting glia formation in NPC cultures. **a** Relative expression of mRNA of the different PKC isozymes under differentiation conditions. mRNA quantification was performed by reverse transcription and real time qPCR and using the Δct method. The mRNA for PKC were measured and normalized to the levels of 18S rRNA. Data are means ± S.E.M. of five independent measurements. **b** Representative fluorescence microphotographs of neurosphere-derived adhered cells transfected with PKCθ siRNA, a combination of PKCθ siRNA and EOF2 or either mock (control). Neuronal cells were identified by the immunocytochemical detection of β-III-tubulin (red); glial cells are identified by the immunocytochemical detection of GFAP (green) and total nuclei were counterstained with DAPI (blue). Scale bar = 50 µm. **c** Graph represents the percentage of total cells (detected by DAPI nuclear staining) that were positive for β-III-tubulin expression after treatments expressed as the percentage of control. Data are the means ± S.E.M. of nine independent measurements (*n* = 9). Statistical analysis: two tailed unpaired Student’s *t* test of each condition compared with control (*p = 0.003 and #*p* = 0.029). **d** Graph represents the percentage of total cells (detected by DAPI nuclear staining) that were positive for GFAP expression after treatment expressed as the percentage of the control. Data are the means ± S.E.M. of nine independent measurements (*n* = 9).

We next study the expression patterns of the classical and novel PKC isozymes in cultures of attached SVZ isolated cells. Classical PKCβ and novel PKCθ, were the most abundant followed by classical PKCα and novel PKCε. Almost undetectable levels of classical PKCγ or novel PKCδ and PKCη were observed (Fig. 4A). Therefore, we analyzed whether blocking the expression of the most abundant novel PKCθ reverted the effect of EOF2. Attached SVZ isolated cells were cultured in the absence of growth factors and transfected with a siRNA to interfere with PKCθ expression as previously described^26^. Cells were left for 72 h in the presence and absence of EOF2 and the percentage of neuroblasts and glial cells was quantified. The elevated percentage of neuroblasts found in the presence of EOF2 was reduced to almost control levels in cultures in which PKCθ expression was inhibited by the siRNA (Fig. 4B, C). EOF2 alone or in combination with PKCθ siRNA had no effect on the percentage of GFAP^+^ cells (Fig. 4B, D).

### NRG1 and ErbB4 expression in brain injuries

In attempt to understand whether this differentiating compound could be used to promote neurogenesis in injuries, we analyzed the expression of neuregulin and its receptor ErbB4 within the perilesional area and in the adjacent SVZ in response to an injury. We performed mechanical cortical injuries in mouse brains and 14 days post injury (dpi) the expression of neuregulin and ErbB4 in the injury and in the ipsilateral SVZ was analyzed by real time qPCR. The expression of NRG1 did not change between the ipsilateral and contralateral side (Supplementary Fig. S2 B) neither within the injury or the SVZ. However, a twofold increase in ErbB4 expression was found in the ipsilateral SVZ (supplementary Fig. S2 A).

### Local infusion of EOF2 in brain injuries enhances the number of neuroblasts

**Fig. 5:**
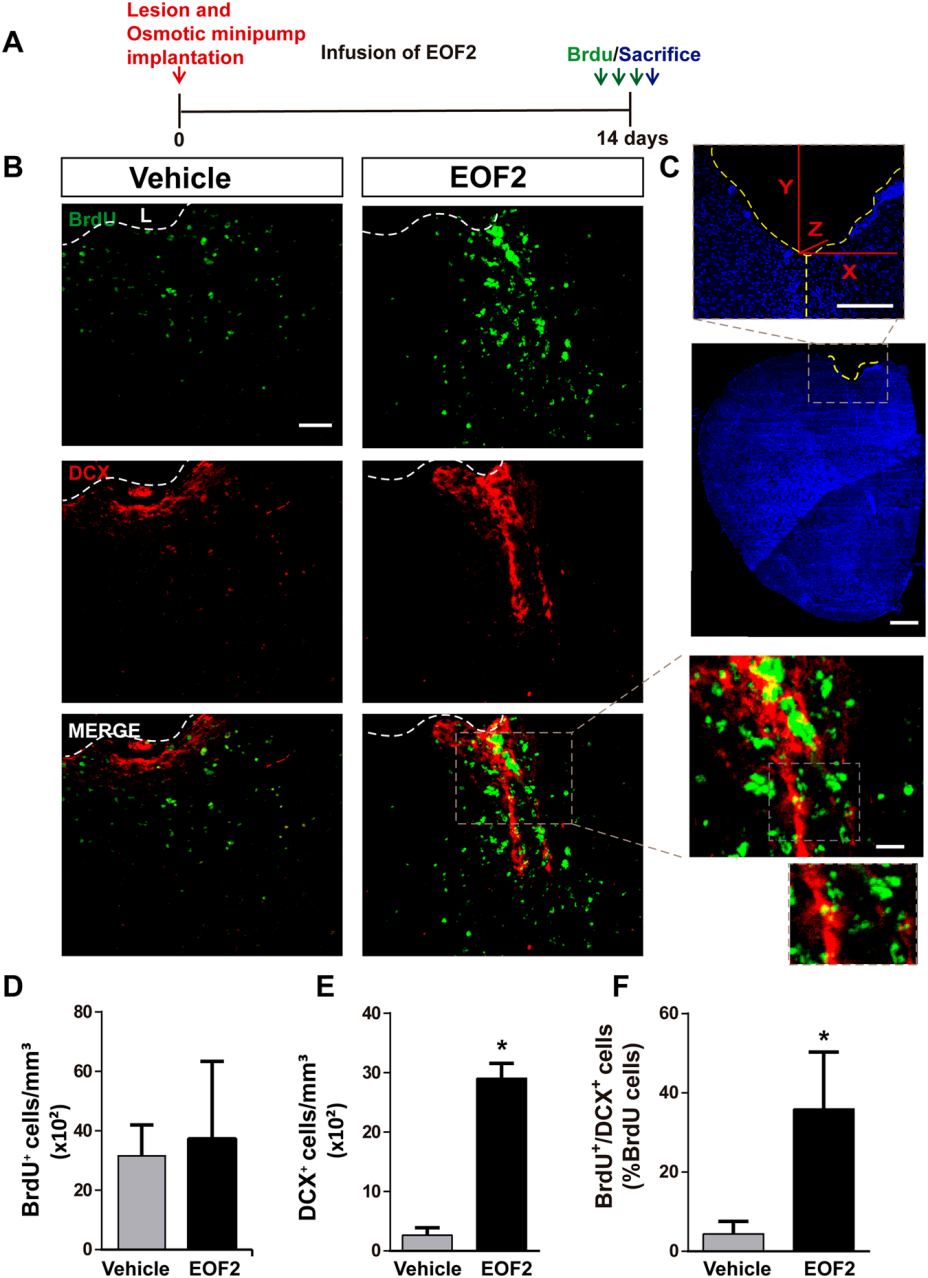
Local administration of EOF2 induces neuronal differentiation in the injured cortex. **a** Scheme of experimental procedures. Mechanical cortical lesions were unilaterally performed in the primary motor cortex of adult mice, and osmotic minipumps were implanted to locally deliver vehicle or EOF2 (5 µM) for 14 days. All mice were intraperitoneally-injected with BrdU on the last day of treatment as described in methods. **b** Representative confocal microphotographs of the area surrounding cortical lesion in mice brain showing immnunodetection for BrdU (upper panels) Doublecortin (DCX; medium panels) and the merged signals (lower panels). Scale bar represent 50 µm in low magnification images and 20 µm in high magnification images. The dotted line indicates the limit of the lesion (L). **c** Microphotograph showing details about the injury and the lesion area. In each section, the positive cells labeled with the different markers were quantified in the peri-lesional area that corresponds to 200 µm-wide band of tissue surrounding the lesion border. Scale bar represent 1 mm in low magnification images and 200 µm in high magnification images. **d** Graph shows the number of proliferating cells labeled with BrdU per mm^3^ in the peri-lesional area of the indicated animal groups. Data shown are the mean ± S.E.M.; *n* = 6 animals per group. **e** Quantification of DCX^+^ (doublecortin) cells per mm^3^ in the peri-lesional area of the indicated animal groups. Data shown are the mean ± S.E.M.; *n* = 6 animals per group. Statistical analysis: *p < 0.0001 in two tailed unpaired Student’s *t* test comparing EOF2 with the control**. f** Graph shows the percentage of BrdU^+^ cells that co-express the neuronal marker DCX in the peri-lesional area of the indicated animal groups. Data shown are the mean ± S.E.M.; *n* = 6 animals per group. Statistical analysis: *p = 0.0320 in two tailed unpaired Student’s *t* test comparing EOF2 with the control.

We next analyzed whether EOF2 infusion in brain injuries facilitated the generation of neuroblasts and neurons within the perilesional area. Mechanical injuries in the motor cortex of mouse brains were performed and EOF2 was locally infused by implanting osmotic minipumps releasing EOF2 or vehicle for 2 weeks. BrdU was injected on the day of sacrifice (Fig. 5A). Treatment had no effect on the extent of the injury or on the size of the perilesional area, in control and treated groups (inter-animal differences: 30 ± 4 × 10^3^ µm^2^/section). The number of BrdU^+^ cells in the perilesional area of mice treated with either vehicle or EOF2 for 14 dpi was similar between the two groups (Fig. 5B, C). Neuroblasts within the peri-lesional area of control mice were hardly visible. Interestingly, the number of neuroblasts increased dramatically in mice treated with EOF2 (Fig. 5B, D–F).

### Local infusion of EOF2 in brain injuries reduces the number of glial cells

**Fig. 6:**
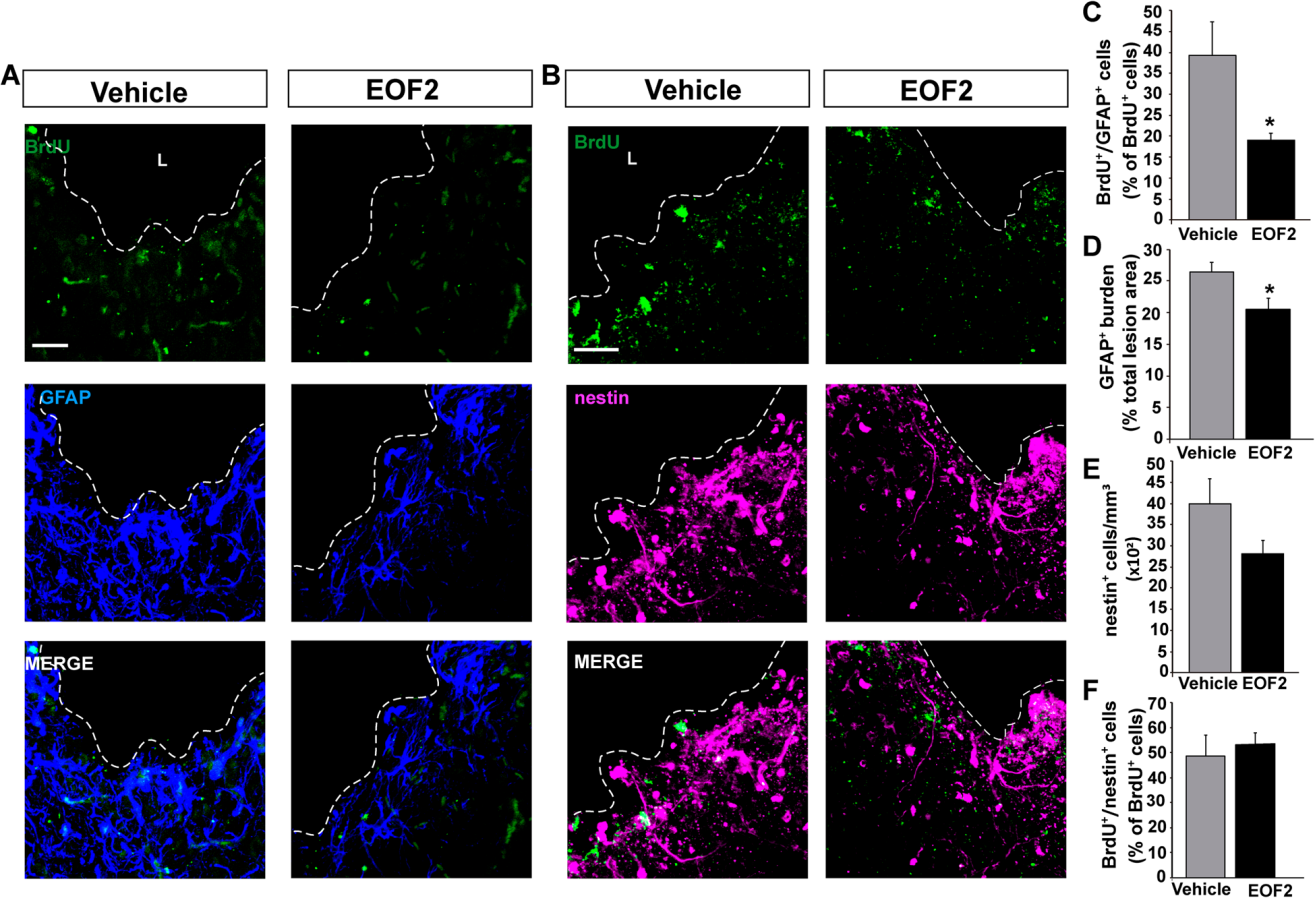
Local administration of EOF2 reduce glial differentiation in the injured cortex. Representative confocal microphotographs of the area surrounding cortical lesion in mice brain processed for the immunohistochemical detection of the glial marker GFAP (**a**) and for the undifferentiated progenitors marker nestin (**b**). Mechanical cortical lesions were unilaterally performed in the primary motor cortex of adult mice, and osmotic minipumps were implanted to locally deliver vehicle or EOF2 (5 µM) for 14 days. All mice were intraperitoneally-injected with BrdU the last day of treatment as described in methods. Scale bar represent 50 µm. The dotted line indicates the limit of the lesion (L). **c** Graph shows the percentage of BrdU^+^ cells that co-express the glial marker GFAP in the peri-lesional area of the indicated animal groups. Data shown are the mean ± S.E.M.; *n* = 6 animals per group. Statistical analysis: *p = 0.0492 in two tailed unpaired Student’s *t* test comparing EOF2 with the control. **d** Graph shows the GFAP burden, representing the glial scar, expressed as a percentage of the total lesion area. Data shown are the mean ± S.E.M.; *n* = 6 animals per group. Statistical analysis: *p = 0.0310 in two tailed unpaired Student’s *t* test comparing EOF2 with the control. **e** Quantification of nestin^+^ cells per mm^3^ in the peri-lesional area of the indicated animal groups. Data shown are the mean ± S.E.M.; *n* = 6 animals per group. **f** Graph shows the percentage of BrdU^+^ cells that co-express the neural precursor marker nestin in the peri-lesional area of the indicated animal groups. Data shown are the mean ± S.E.M.; *n* = 6 animals per group.

We next analyzed the number of BrdU^+^ cells that expressed the glial marker GFAP in both control and EOF2 treated mice, as well as the area occupied by glial cells as a measure of gliosis and glial scar. Noticeable, the GFAP burden was significantly reduced in injuries treated with EOF2 compared with controls (Fig. 6A–D). Accordingly, the percentage of BrdU^+^ cells that co-localized with the glial cell marker GFAP was also reduced in treated injuries (Fig. 6A–C). This suggested a role for EOF2 in reducing gliosis around the injured area (Fig. 6A, B). No effect of the treatment was observed on the number of nestin^+^ undifferentiated progenitors or in its proliferation rate (Fig. 6B, E, F).

### Local treatment of injuries with EOF2 did not affect neurogenesis in the SVZ or DG

It was then tested whether local treatment with EOF2 affected neurogenesis in the SVZ and DG. We analyzed BrdU^+^, BrdU^+^/DCX^+^ BrdU^+^/GFAP^+^, and BrdU^+^/nestin^+^ cells in both the SVZ and DG. As expected, in response to the injury, the number of BrdU^+^ cells was significantly increased in the ipsilateral SVZ (supplementary fig. S3 A–F) and DG (supplementary fig. S4 A–F) of control and EOF2-treated mice compared with the contralateral areas, indicating the presence of a proliferative response to the injury. However, no effect of EOF2 was observed on the proliferation of progenitor cells and their progeny in the two neurogenic niches (supplementary figs. S3 and S4 A–F).

### Intranasal administration of EOF2 facilitates neuroblast migration towards the perilesional area

**Fig. 7:**
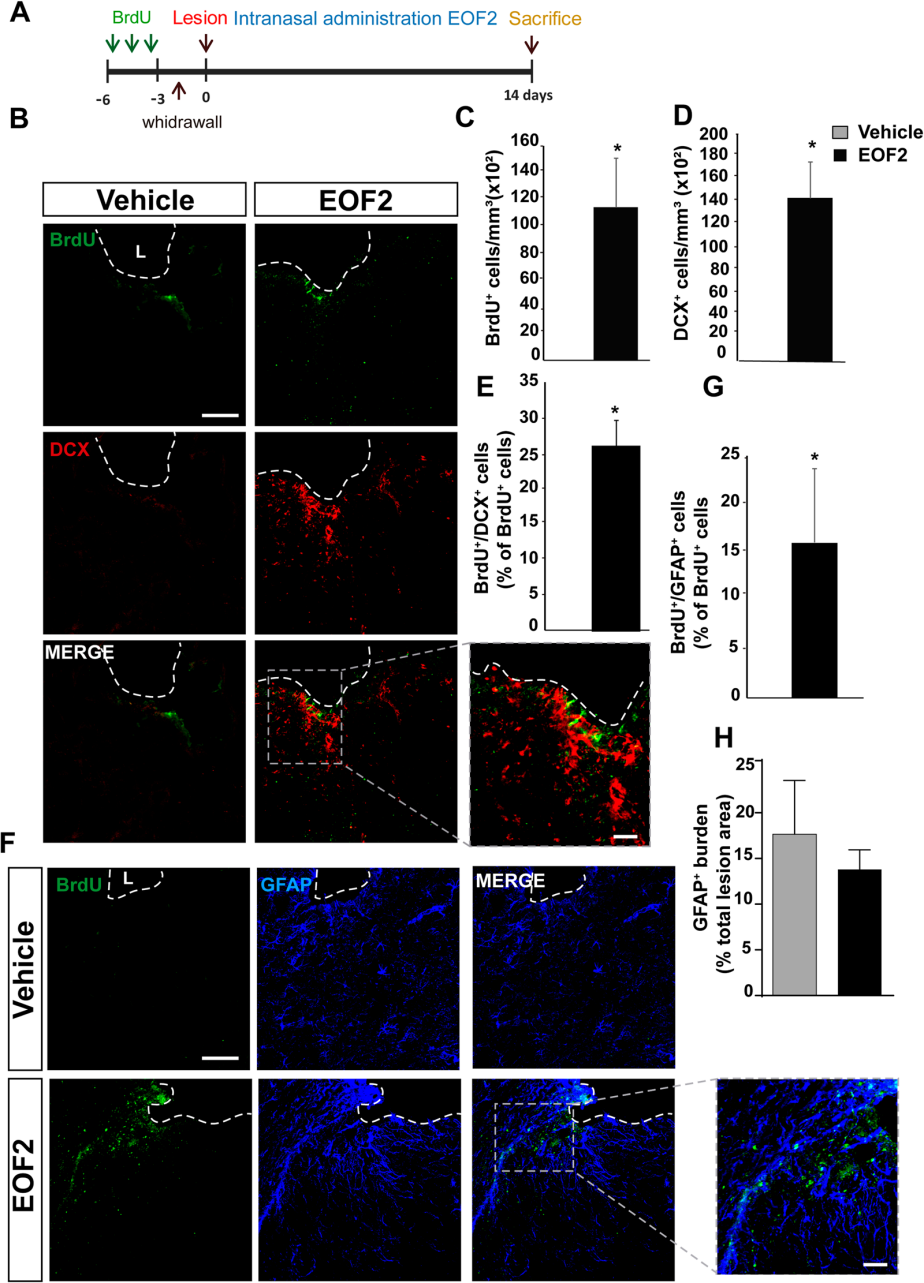
Intranasal administration of EOF2 induce neuroblast migration from the neurogenic regions to the damage area. **a** Scheme of BrdU administration. Experimental procedure followed to label proliferating neural precursors with BrdU exclusively in neurogenic niches and not in the injured area: mice received BrdU injections on days 6, 5 and 4 before performing the cortical injury; then we waited three more days to allow for complete withdrawal of BrdU from the animal organism. Lesion was performed on day 0. Intranasal EOF2 (5 µM) or only vehicle was administered for 14 days. **b** Representative confocal microscopy images of the injured cortex of adult mice after bearing cortical lesions and the intranasal administration of EOF2 or only vehicle processed for the immunohistochemical detection of the proliferation marker BrdU and the neuroblast marker doublecortin (DCX). The dotted line indicates the limit of the lesion (L) and the scale bar represent 100 µm in the low magnification pictures and 50 µm in the high magnification picture. **c** Graph shows the number of proliferating cells marked with BrdU per mm^3^ in the peri-lesional area of the indicated animal groups. Data shown are the mean ± S.E.M.; *n* = 6 animals per group. Statistical analysis: *p = 0.0377 in two tailed unpaired Student’s *t* test comparing EOF2 with the control. **d** Quantification of DCX^+^ cells/mm^3^ in the peri-lesional area of the indicated animal groups. Data shown are the mean ±S.E.M.; *n* = 6 animals per group. Statistical analysis: *p = 0.0012 in two tailed unpaired Student’s *t* test comparing EOF2 with the control. **e** Percentage of BrdU^+^ cells that co-expressed DCX in the peri-lesional area. Data shown are the mean ± S.E.M.; *n* = 6 animals per group. Statistical analysis: *p = 0.0100 in two tailed unpaired Student’s *t* test comparing EOF2 with the control. **f** Representative confocal microscopy images of the injured cortex of adult mice in the previously indicated groups processed for the immunohistochemical detection of the proliferation marker BrdU and the glial marker GFAP. The dotted line indicates the limit of the lesion (L) and the scale bar represent 100 µm in the low magnification pictures and 50 µm in the high magnification picture. **g** Percentage of BrdU^+^ cells that co-expressed GFAP in the peri-lesional area. Data shown are the mean ± S.E.M.; *n* = 6 animals per group. Statistical analysis: *p = 0.0214 in two tailed unpaired Student’s *t* test comparing EOF2 with the control. **h** Graph shows the GFAP burden within the peri-lesional area, expressed as a percentage of the total lesion area. No statistically significant differences were found. Data shown are the mean ± S.E.M.; *n* = 6 animals per group. Statistical analysis: *p* = 0.4464 in two tailed unpaired Student’s *t* test comparing EOF2 with the control.

In order to test whether neuroblast found in EOF2 treated mice had migrated from the adjacent SVZ, we had to find a method of administration that allowed EOF2 to reach neurogenic regions. In addition, used a BrdU labeling approach that allowed BrdU to incorporate in proliferating cells of the SVZ before performing the injury. Thus, healthy mice were injected with BrdU for three consecutive days and left for another 3 days for the BrdU to be cleared. By using this strategy, only proliferating cells in neurogenic niches were labeled with BrdU. Then, mice were injured and treated for 14 days with daily intranasal administrations of EOF2 or vehicle (Fig. 7A). Mice were sacrificed 14 dpi and the co-localization of BrdU with DCX, and GFAP was quantified. Interestingly, BrdU^+^ cells were only detected within the perilesional area of mice treated with EOF2 (Fig. 7B, C). Also, we were able to find a significant amount of DCX^+^ neuroblasts that were not present within the perilesional area of control mice (Fig. 7B, D). BrdU^+^ cells were GFAP^+^ or DCX^+^ (Fig. 7B, E–G). Despite the presence of BrdU^+^/GFAP^+^ cells in treated mice, no effect on the GFAP burden was observed in treated mice compared with control (Fig. 7H). The analysis of BrdU^+^ cells in the SVZ shows a large reduction in the number of BrdU^+^ cells in the ipsilateral SVZ of both control and EOF2 treated mice in comparison with their contralateral SVZ (supplementary fig. S5). Moreover, EOF2 treated mice showed a smaller number of BrdU^+^ cells in the SVZ than their control counterparts (supplementary fig. S5). In addition, we have observed that neuroblasts migrated from the SVZ of EOF2 treated mice towards the injury through organized migratory pathways that described a track from the SVZ towards the injury through the corpus callosum reaching the lesioned area (supplementary fig. S6). This was not observed in control mice.

**Fig. 8:**
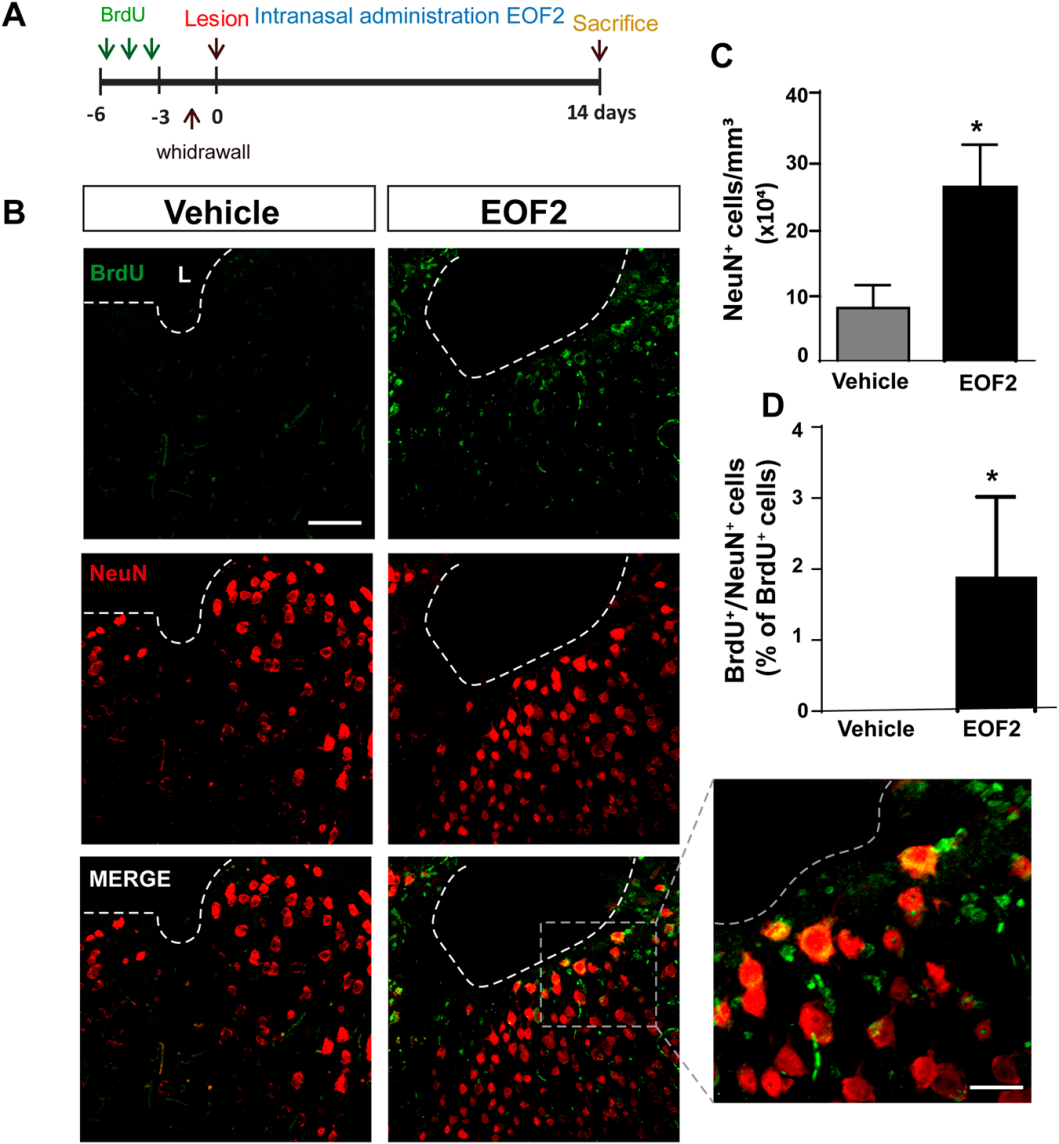
Intranasal administration of EOF2 induces neuronal differentiation in the peri-lesional area after brain injury. **a** Scheme representing the experimental procedure followed to label proliferating neural precursors with BrdU exclusively in neurogenic niches and not in the injured area: mice received BrdU injections on days 6, 5, and 4 before performing the cortical injury; then we waited three more days to allow for complete withdrawal of BrdU from the animal organism. Lesion was performed on day 0. Intranasal administration of EOF2 (5 µM) or only vehicle was performed for 14 days. **b** Representative confocal microscopy images of the injured cortex of adult mice after bearing cortical lesions and the intranasal administration of EOF2 or only vehicle. The dotted line indicates the limit of the lesion (L) and the scale bar represent 100 µm in the low magnification pictures and 20 µm in the high magnification pictures. **c** Graph shows the number of neuronal cells marked with NeuN per mm^3^ in the peri-lesional area of the indicated animal groups. Data shown are the mean ± S.E.M.; *n* = 6 animals per group. Statistical analysis: *p = 0.0339 in two tailed unpaired Student’s *t* test comparing EOF2 with the control. **d** Percentage of BrdU^+^ cells that co-expressed the neuronal marker NeuN in the peri-lesional area. Data shown are the mean ± S.E.M.; *n* = 6 animals per group. Statistical analysis: *p = 0.0411 in two tailed unpaired Student’s *t* test comparing EOF2 with the control.

In addition, only EOF2-treated mice showed mature NeuN^+^ neurons that had incorporated BrdU within the perilesional area (Fig. 8A, B–D). Accordingly, a larger number of mature neurons were quantified within the perilesional area of EOF2-treated mice 14 dpi (Fig. 8B, C). We next use a longer treatment (28 days) that allowed neuroblasts to further differentiate maintaining the BrdU labeling protocol. We analyzed the number of BrdU^+^ cells within the perilesional area after a 28-day treatment and assessed their capacity to differentiate into mature neurons of different phenotypes. We found that the number of BrdU^+^ cells 28 dpi was reduced by 50% (supplementary fig. S7 A–C) compared with 14 pdi. Almost 35% of the BrdU^+^ cells co-localized with Choline acetyl transferase (ChAT^+^) (supplementary fig. S7 A, D) and around 5% co-localized with parvalbumin (supplementary fig. S7 B–D) indicating that some of the new neurons had differentiated into cholinergic and GABAergic neurons.

## Discussion

We have studied in here the neurogenic effect of a small molecule, the diterpene with lathyrane skeleton EOF2. Unlike other lathyranes, EOF2 does not promote proliferation of NPC^22^. However, we have demonstrated that it specifically facilitates novel PKC-mediated NRG1 release, without affecting TGFα release. This molecule promotes neuronal differentiation of NPC in vitro in a novel PKC dependent manner. Moreover, EOF2 in vivo enables migration of neuroblasts from neurogenic areas towards an injury leading to neuroblast and neuron enrichment in damaged brain regions.

### Targeting neuregulin release to promote neurogenesis in injuries

Previous reports have demonstrated the role of individual PKC isozymes as mediators of different steps in the neurogenic process^27,28^, some of them highlighting the role of non-tumor promoting diterpenes as PKC activators that promote neurogenesis in neurogenic niches^22,29^. However, neurogenic signaling molecules behave differently depending on the niche. This is the case of TGFα, a growth factor that promotes neurogenesis in neurogenic niches^30^ while inducing gliogenesis and impairing neuroblast migration in injuries^7,11^. Thus, a key step to facilitate neurogenesis in injuries is the introduction of neurogenic cues to counteract the gliogenic ones generated by the injury. In this work, we have focused on neuregulins, which are expressed in neuroblasts, and activate TrkA receptors on neighbor cell types. The pathways associated to these receptors promote neuroblast survival^31^. Also, neuregulin-mediated activation of ErbB4 receptors facilitates NSC proliferation and neuroblast migration^20,21^. We searched for a compound able to deliver neuregulins to the injured brain tissue. We chose to study the effect of EOF2 because of its lack of effect on stimulating NPC proliferation in a classical PKC dependent manner^22^ and its similarity to other classical PKC activating compounds (supplementary fig. 8). According to the report of Newton 2018^32^, the affinity of novel PKC isozymes for DAG and therefore for this type of compounds is higher than that of classical PKC isozymes suggesting the possibility that EOF2 was a novel PKC activating compound with no capacity to activate the classical.

PKC isozymes participate in the release of ligands that activate ErbB receptors^25^. These ligands are synthesized and transported to the plasma membrane as transmembrane pro-ligands. The active soluble ligand is proteolyzed from the pro-ligand and released to the extracellular medium in a reaction catalyzed by enzymes of the ADAM family, particularly ADAM17^15^. The selectivity of ADAM17 for its substrates depends on phosphorylation reactions on the pro-ligands catalyzed PKC kinases^18,19^. Thus, phosphorylation of TGFα, HB-EGF, amphiregulin and other EGFR ligands by classical PKC determines their secretion, whereas, phosphorylation of NRG1 by novel PKC isozymes facilitates its release^18,19,25^. We used fusion proteins in which NRG1 and TGFα were fused to two fluorescent probes. Similar fusion proteins had previously been used for this particular purpose^25^. As time-lapse imaging fluorescence experiments show, EOF2 induces the shedding of NRG1 in HEK293T transfected cells, in a time dependent manner. The release of neuregulin to the extracellular medium was still partially observed in the presence of a classical PKC inhibitor, which at the concentration required to inhibit cPKC by 80%, partially inhibits novel PKC by 30–40%^33^. This indicates that the inhibition of classical PKC does not impair NRG1 release and suggests a role for novel PKC isozymes in the EOF2 stimulated NRG1 release. Unfortunately, commercially available specific inhibitors of novel PKC could not be used to definitely demonstrate the role of novel PKC isozymes in EOF2-mediated NRG1 release. EOF2 did not induce TGFα release to the extracellular medium, indicating that EOF2 specifically facilitated NRG1 release. These results agree with previous studies showing that PKC-δ is required for NRG1 cleavage, and that PKC-δ phosphorylation of serine 286 in the neuregulin cytosolic domain is essential for induced neuregulin cleavage^18^. Interestingly, other reports show that classical PKC activation is required for PMA-induced cleavage of all EGFR ligands^19^.

### Novel PKC-mediated differentiation of NPC into neuroblasts

EOF2 promoted neuronal differentiation of NPC in vitro in cultures of SVZ isolated cells. This effect correlated with a reduction in the percentage of nonviable cells, suggesting an effect of EOF2 on neuroblast survival and in neuroblasts differentiation. Considering the role of neuregulins in maintaining neuroblasts survival^31^, it would be possible that EOF2-mediated neuregulin release facilitated neuroblasts survival in our cultures. However, an effect on NPC differentiation towards a neuronal fate is also possible, and a combination of both effects might be the reason for the elevated number of neuroblasts. The effect of EOF2 was reverted by blocking the expression of the most abundant novel PKC isozyme found in our cultures, PKCθ. This result represents another evidence that supports the role of EOF2 in novel PKC activation, and neuronal differentiation probably mediated by neuregulin release.

### Enabling neuronal replacement in injuries with EOF2

Previous studies describe a response of neurogenic niches to cortical and spinal cord injuries^34–38^. Most of these studies show a neurogenic response of the DG and SVZ together with the absence of newly generated neuroblast within the perilesional area in control conditions. Interestingly, inhibiting ADAM17-mediated release of TGFα resulted in a neurogenic response within the perilesional area that produced a large number of new neurons^8^. In agreement with these studies, we show in here that injuries of control mice treated with vehicle showed none or a very small number of neuroblasts. The importance of our work is that we find a considerable number of newborn neurons in injuries of mice treated with EOF2. Previous results have identified neuroblasts migrating from the SVZ towards the site of injury through the corpus callosum. However, in the absence of treatments these neuroblasts never reached the injured site^8^. This indicated that signaling molecules that lead neuroblasts towards the injury, need to be released—as described in Comte et al. 2011, for Galectin-3—to maintain motility from the SVZ to the olfactory bulb^39^. Since neuregulins play a role in neuroblast migration and survival it was reasonable to test the effect of EOF2 on migration finding an administration method that allowed the continuous administration of the compound avoiding local damage and allowing a more extensive delivery of EOF2, reaching the DG and SVZ. We used intranasal delivery, a method that has been successfully used in previous works^40–42^. Detection of migrating cells was possible by using a BrdU labeling strategy in which BrdU was administered to the mice 6 days prior to inducing the injury. Using this strategy, we have demonstrated that the presence of the injury induces migration of BrdU^+^ cells from the SVZ but only in mice treated with EOF2 BrdU^+^ cells migrate from the SVZ reaching the injury. Moreover, in treated mice a considerable number of DCX^+^ neuroblasts and NeuN^+^ neurons that co-localize with BrdU were found within the perilesional area 14 dpi indicating that these neuroblasts had migrated from the SVZ. The presence of neuroblasts describing a track from the SVZ through the corpus callosum and cortex and reaching the lesioned area in treated mice supports this fact (supplementary fig. S6). An attempt of neuroblast migration was observed from the SVZ of control mice, however, no neuroblasts were found within the corpus callosum, the cortex or around the perilesional area in these mice. Neuroblasts left the SVZ and reached the corpus callosum without migrating through it towards the injury (supplementary fig. S6). After a longer 28-day treatment with EOF2 around 50% of the BrdU^+^ cells found 14 dpi survived. At this stage, a percentage of the BrdU^+^ cells had differentiated into mature cholinergic neurons and a few of them differentiated into parvalbumin^+^ GABAergic neurons. The rest of BrdU^+^ cells, not labeled with ChAT or parvalbumin, may be neuroblasts, undifferentiated progenitors, glial cells, or they may have developed a different neuronal phenotype. In summary, all this indicates that EOF2 facilitated migration of neuroblasts and that these neuroblasts differentiated into mature neurons. Similar results have been obtained upon reduction of TGFα release by ADAM17 inhibition^8^. Therefore, it is feasible that the capacity of EOF2 to favor neuregulin release over TGFα is promoting neurogenesis in injuries.

## Conclusion

In conclusion, we are reporting in here the discovery of EOF2 as a new non-tumorigenic specific activator of novel PKC isozymes, with the capacity to induce neurogenesis in brain injuries. We have unraveled its mechanisms of action mediated by its capacity to activate novel PKC releasing neuregulin. In addition, we have used an effective, noninvasive intranasal administration method of this compound for its effective delivery in the mouse brain promoting neuronal replacement in injuries. Our work highlights the role of novel PKC, as targets and for an effective treatment to compensate neuronal loss in damaged brain areas, being of special relevance in the development of new drugs to use in brain injury regeneration strategies.

## Materials and methods

### Reagents

The lathyrane EOF2 3,8,12-tri-*O*-acetyl-7-*O*-(4-methoxyphenyl) acetylingol; CAS number 2230806–06–9; was isolated and purified in our laboratory, as described previously^43,44^. Briefly, purification by semipreparative HPLC was performed with a Hitachi/Merck L-6270 apparatus equipped with a differential refractometer detector (RI-7490). A LiChrospher® Si 60 (10 μm) LiChroCart® (250 mm × 10 mm) column was used in isolation experiments. Silica gel (Merck) was used for column chromatography. TLC was performed on Merck Kieselgel 60 F254, 0.25 mm thick. Infrared spectra were recorded on a FT-IR spectrophotometer and reported as wavenumbers (cm^−1^). ^1^H and ^13^C NMR measurements were obtained on a 400 MHz spectrometer with SiMe_4_ as the internal reference. Chemical shifts were referenced to CDCl_3_ (δ_H_ 7.25, δC 77.0), NMR assignments were made by a combination of 1D and 2D techniques. Multiplicities are described using the following abbreviations: s = singlet, d = doublet, t = triplet, q = quartet, m = multiplet, and br = broad. High-Resolution Mass Spectrometry was performed with a QTOF mass spectrometer in positive ion ESI mode.

The commercial PKC activators PMA (13-*O*-acetyl-12-*O*-tetradecanoylphorbol, also known as TPA) and prostratin (13-*O*-acetyl-12-deoxyphorbol) were both purchased from Sigma-Aldrich (St. Louis, MO, USA). The general PKC inhibitor bisindolylmaleimide I (also known as GF109203X, GFX or Gö6850) and the classical PKC inhibitor Gö6976 were from Calbiochem (Millipore, Billerica, MA, USA), and were added to the cells at final concentrations of 5 µM and 1 µM, respectively. Stock solutions of all PKC interacting compounds were prepared in DMSO and pre-diluted in culture medium before addition to cell cultures. When cultures were co-treated with PKC inhibitors and activators, PKC inhibitors were added to the cells 30 min before addition of PKC activators. Specific small interference RNAs (siRNAs) were from Thermo Scientific Dharmacon (Lafayette, CO, www.dharmacon.com).

Other products, unless otherwise indicated, were purchased from Sigma-Aldrich (St. Louis, MO, USA).

### Animal subjects

CD1 male mice were used throughout this study. Animals were housed under controlled conditions of temperature (21–23 °C) and light (LD 12:12) with free access to food (AO4 standard maintenance diet, SAFE, Épinay-sur-Orge, France) and water. Care and handling of animals were performed according to the Guidelines of the European Union Council (2010/63/EU), and the Spanish regulations (65/2012 and RD53/2013) for the use of laboratory animals. All studies involving animals are reported in accordance with the ARRIVE guidelines for reporting experiments involving animals^45,46^.

Only male mice were used in this work. The total number of animals used was: 24 2-month old adult mice and 150 7-day old pups. The number of animals used in each experiment was determined based on previous studies^22,29,47^.

Adult male mice were randomized during the first week after birth by cross fostering and male were used at the age of 2 months.

The protocol used has been authorized by the Ethics Committee of the “Consejería de Agricultura, Pesca y Desarrollo Cultural de la Junta de Andalucía”, Spain with the approval number 30/03/2016/038.

### SVZ cell isolation and culture

NPC were isolated from the SVZ of 7-day postnatal (P7) mice following the same procedure described before^48^, and were cultured as described elsewhere^49^. The sacrifice of P7 mice was done by decapitation without anesthesia, and six pups were used for each independent culture. EGF (20 ng/ml, from GIBCO) and bFGF (10 ng/ml; from PeproTech, Frankfurt, Germany) were used for culture expansion, but only bFGF was present in most experimental settings, unless otherwise indicated.

### Cloning of human neuregulin and TGFα cDNA fused to GFP and Cherry

Full-length cDNA encoding the membrane-bound isoform of human pro-neuregulin-1 β1-type (NRG1, NCBI reference sequence: NP_039250.2) with mCherry cDNA inserted between nucleotides 93 and 94 of NRG1 open reading frame was cloned into pEGFP-N1 to add EGFP cDNA to the 3′ end. Construct was synthesized by GeneCust (Boynes, France) to generate the mCherry-NRG1-GFP construct. The mCherry-TGFα-GFP construct containing the human transforming growth factor alpha (TGFA, NCBI reference sequence: NM_003236.4), containing mCherry cDNA between nucleotides 126 and 127 of TGFA was built using the same strategy and synthesized by GeneCust (Boynes, France).

### HEK293T cultures and transfection

HEK293T cells were obtained from ATCC (Manassas, VA, USA). They were cultured in DMEM at 37 °C and 5% CO2 (Thermo Fisher Scientific, Inc., Rockford, IL, USA), supplemented with fetal bovine serum (10%), 1× GlutaMA^TM^-I (Thermo Fisher Scientific, Inc., Rockford, IL, USA) and penicillin/streptomycin (1%). Cells were passaged, seeded, and allowed to attach for 24 h. Lipofectamine 2000 (Invitrogen; Carlsbad, CA, USA) was used for transfection of these plasmids. Afterwards, medium was changed to eliminate Lipofectamine. After an overnight incubation, the cells were starved for at least 30 min in serum-free Fluorobrite DMEM (Thermo Fisher Scientific) containing 1% P/S, 0.25% bovine serum albumin, and 1× GlutaMAX^TM^-I. and cells were used either in time-lapse or fluorescence experiments. Control experiments were performed to make sure that the 3.5 h serum starvation did not affect cell viability. The percentages of viable transfected cells in serum starved vs non-starved were 95.12 ± 0.73 vs 94.97 ± 0.5, indicating that serum deprivation for 3.5 h did not exert an effect on cell viability.

### Cell viability assays

Cells from neurospheres were seeded at a density of 20000 cells/mL, and cultured in the absence or presence of specific treatments and left for 72 h. Then, cells were detached and mixed with trypan blue [0.04% wt/vol in phosphate-buffered saline (PBS)]. Hek293 cells were seeded at a density of 200000 cells/mL and cultured as indicated in the above section. Then cells were detached from the surface and mixed with trypan blue [0.04% wt/vol in phosphate-buffered saline (PBS)]. Nonviable cells (those including the trypan blue dye) and viable cells (those excluding trypan blue) were counted using a hemocytometer under an inverted microscope and were expressed as percentage of the total number of cells. Samples were coded and blinded quantifications were done in five independent experiments; each of these five independent experiments was performed in triplicate. Experiments were performed and quantified by different persons.

### In vitro time-lapse experiments

Transfected HEK293T cells were plated in 35 mm high µ–dishes (Ibidi, Munich, Germany). Cells were treated with EOF2 or inhibitors, as described in the results and figure legends. Time-lapse assays were performed with a Zeiss Axio Observer.Z1-Inverted Microscope, using a plan-apochromat 40× / 0.95 Korr M27 air objective lens. Images of transfected cells were obtained every 1 min. Captured images were processed using ZEN lite software and the efficiency of NRG1 and TGFα cleavage determined by analyzing the mCherry/GFP fluorescence intensity over the entire cell areas. The mCherry/GFP ratios were calculated and normalized to the average ratio measured before stimulation with EOF2 using the Microsoft Excel software. Ratiometric images were built using ImageJ software, after background subtraction, the mCherry/GFP image was calculated dividing mCherry channel by the GFP channel. For each pixel, a pseudocolor scale is used for coding the ratio.

### Fluorescence analysis of mCherry-fused TGFα or neuregulin in the culture medium of HEK293T

Transfected HEK293T cells were plated in Costar® 12-well cell culture microplate in 1 mL volume of culture medium. Upon treatment with the EOF2 and/or inhibitors, 50 μL aliquots of culture medium were removed at 30, 60, and 180 min and specific mCherry fluorescence was measured using the culture medium of non-transfected cells as blank. Then, cells were washed with PBS, scratched from the surface of the plates and GFP fluorescence signal of the whole cells for each condition was determined using non-transfected cells as blank. Fluorescence of non-treated transfected cells was used as control. All fluorescence experiments were done using Costar® 96-well black polystyrene plates and a BioTek™ Synergy™ Mx Monochromator-Based MultiMode Reader. Interference fluorescence from the culture media was minimized by the optimization of the detection parameters: mCherry: excitation 587 nm, emission 610 nm; GFP: excitation 488 nm, emission 510 nm. Positive controls of TGFα and NRG1 shedding, after classical PKC activation were done previously using PMA and inhibitors.

### PKC kinase activity assay

Neurospheres were disaggregated and 20,000 single cells were seeded per well. Treatments (5 μM EOF2 or vehicle) were added for 1 h prior to cell centrifugation (200 g, 5 min) and lysis. Inhibitors were added 30 min before the addition of the treatments. Protein content was measure in the lysates using the BCA method (Thermo Fisher Scientific, Rockford, IL, USA), and 1.5 μg of crude protein was used per assay. The amount of PKC kinase activity was measured in each sample using the PKC Kinase Activity Assay Kit (Abcam, Cambridge, U.K.; cat. No. ab139437), following the manufacturer’s instructions. Positive controls (20–60 ng of purified active PKC supplied by the kit) and blanks (diluent only) were included in each independent determination. Blanks were subtracted from measurements before comparisons were made.

### Cell culture adhesion, treatment, and transfection

Neurosphere cells were centrifuged, resuspended in defined medium with growth factors, and seeded. Cell transfection with specific siRNAs was performed 18 h after seeding; for this, cells were changed to antibiotic-free medium and transfected using Lipofectamine 2000, following the manufacturer’s instructions. Lipofectamine was removed 6 h later, EOF2 was added and cells were maintained for 48 additional hours before being fixed for immunocytochemistry.

### Immunocytochemistry

Immunostaining for GFAP and β-III-tubulin was performed as previously described^29^. The primary antibodies used for this were: mouse monoclonal anti-βIII-tubulin (1:1000, Cell Signaling Technology, Boston, MA, USA) and rabbit polyclonal anti-GFAP (1:3000, Dako, Hamburg, Germany). The secondary antibodies used were: donkey anti-rabbit IgG labeled with AlexaFluor® 488 (1:1000) and donkey anti-mouse IgG labeled with AlexaFluor® 594 (1:1000), from Invitrogen (Carlsbad, CA, USA). Samples were coded before immunostaining and blinded quantifications of labeled cells were done in nine independent experiments. Experiments were performed and quantified by different individuals to avoid subjective bias during the quantification process. Original fluorescence colors have been changed in some of the pictures shown, using the ImageJ software, to enhance visualization of markers.

### RNA isolation, reverse transcription and real-time quantitative PCR

Total RNA isolation from differentiated cells, the injured and the intact cortex, reverse transcription and RT-qPCR to relatively quantify the expression of the different isozymes of PKC were performed as previously described^7^. The housekeeping transcript used was 18S rRNA. All PCR reaction within each experiment was run in duplicates. Amplification specificity was confirmed by melting-curve analysis of the PCR products. The relative expression of each mRNA was calculated as 2^−ΔCt^, where ΔCt = Ct (target mRNA)−Ct (18S). No signal was detected in non-template or non-RT controls.

Primer sequences (5´-3´) and annealing temperatures were the following: for detecting expression of mouse mRNA levels in neuroespheres we used the following primers: PKCα, FW: TGAATCCTCAGTGGAATGAGT, RW: GGTTGCTTTCTGTCTTCTGAA, 55 °C; for PKCβ, FW:CCCGAAGGAAGCGAGGGCAATGAAG, RW:AGTTCATCTGTACCCTTCCGCTCTG, 63 °C; for PKCγ, FW:TGAGAGAGTGCGGATGGGCCCC, RW:GCAGGCGTCCTGGGCTGGCACC, 65 °C; for PKCδ, FW:GAGGCCTTGAACCAAGTGACCC, RW:CTTGCCATAGGTCCAGTTGTTG, 57 °C; for PKCε, FW:CCCATCTGAAGACGACCGATCC, RW:CGGTTGTCAAATGACAAGGCC, 60 °C; for PKCθ, FW:CCATGTCACCGTTTCTTCGAATC, RW:TCTGCCCATTTTCTGATTCC, 56 °C; for PKCη, FW:ATGGCCACGTACCTGAGGCAGC, RW:GGACGACGCAGGTGCACACTTGG, 65 °C; for PKCλ, FW:CGTTGGGAGCTCTGACAATC, RW:ACCTGCTTTTGCTCCATCATG, 55 °C; for PKCζ, FW:AGGAGAAGAGTACGGGTTCAGC, RW:GTGTTCATGTCAGGGTTGTCCG, 57 °C.

For mouse neuregulin and ErbB4 in injuries and SVZ we used NRG1, FW: CGCTGTTCTGGTCTCATCCG, RW: GCGGTGGAGTGGAGTGTAAG, 54 °C; for ErbB4, FW: TACCTCCTCCCATCTACACATCC, RW: CCTCTGGTATGGTGCTGGTTG, 57 °C.

The primers used for detection of human PKC isozyme mRNAs from HEK293 are described supplementary table T1.

### Mechanical lesions in brain cortex

Unilateral cortical lesions were performed in the cortex of the right hemisphere of adult mice anesthetized with an intraperitoneal injection of a 100 mg/kg ketamine and 20 mg/kg xylazine cocktail. Animals were placed in a stereotaxic frame (Kopf Instruments), and a small craniotomy was performed at +1.4 mm rostral and +1.5 mm lateral to Bregma. A controlled mechanical lesion was performed in the underlying primary motor cortex, using a manually driven drill (0.7 mm diameter) that was allowed to penetrate 1 mm below the bone surface. These injuries reached the corpus callosum without damaging it.

### Studies describing neurogenic responses in mechanical lesions

Mice were injured using the procedure mentioned above, and were sacrificed at 14 days post-injury (dpi). Mice were given three intraperitoneal injections of BrdU (70 mg/kg each) separated by 3 h intervals on the day of sacrifice. Mice were sacrificed by brain perfusion, and brains were processed for post-mortem studies as described below. In the same surgical acts in which cortical lesions were performed, Alzet osmotic mini-pumps (Charles Rivers Barcelona, Spain) were implanted subcutaneously in 12 animals and connected to infusion cannulas (brain kit II, Alzet) whose tips were placed 0.5 mm deep into the lesion, allowing a continuous delivery of a 5 µM solution of EOF2 in PBS (containing 0.4% DMSO) or vehicle (six mice received EOF2 and six mice received vehicle). Treatments lasted for 14 days.

### Studies describing migration towards mechanical lesions

In a different set of experiments, aimed to study the migration of progenitors from neurogenic regions towards the injured area, mice received BrdU injections 6, 5, and 4 days before the injury was performed. This paradigm allowed for substantial clearance of BrdU and, therefore, it allowed BrdU labeling of NPC in the SVZ but not locally in the injured tissue. These mice (vehicle; *n* = 6 and EOF2-treated; *n* = 6) were sacrificed on day 14 after injury. Starting the day of injury, animals were treated with daily intranasal administrations of a 5 µM solution of EOF2 or vehicle, until they were sacrificed 14 dpi (Figs. 7, 8).

### Intranasal administration of EOF2

EOF2, was delivered intranasally as previously described^40–42^. Treatments were administered manually while the animal was placed in a standing position with an extended neck as previously described^42^. 18 µL of each solution 5 µM EOF2, or vehicle was delivered over both nasal cavities alternating 3 µL/each using a micropipette. Mouse was maintained in such position for ten additional seconds to ensure all fluid was inhaled.

In all experiments, mice were coded, treatment (vehicle or EOF2) was assigned randomly to code numbers and applied. In addition, blind quantifications were performed to avoid subjective biases.

### Immunohistochemistry

Brain processing and immunohistochemical detection of the proliferation marker BrdU, the astrocyte and neural stem cell marker GFAP, the neural progenitor cell marker nestin, the early neuronal differentiation marker doublecortin (DCX), and the mature neuron maker NeuN were performed as previously described^49^.

Primary antibodies used were mouse monoclonal anti-BrdU (1:100) from Dako (Hamburg, Germany) or rat monoclonal anti-BrdU (1:100) from Abcam (Cambridge, UK), mouse polyclonal anti-GFAP both from Cell Signaling (Beverly, MA, USA), goat polyclonal anti-DCX (1:500), goat polyclonal anti-nestin (1:500), and mouse monoclonal anti-NeuN (1:100) all of them from Abcam (Cambridge, UK); goat anti-ChAT, polyclonal, 1:100, Merk Millipore (Billerica, MA, USA) mouse anti-parvalbumin, monoclonal, 1:100, Merk Millipore (Billerica, Ma, USA). Secondary antibodies used were Alexa Fluor 488 donkey anti-mouse, Alexa Fluor 594 donkey anti-mouse, Alexa Fluor 405 goat anti-mouse, Alexa Fluor 594 donkey anti-rat, Alexa Fluor 488 donkey anti-rabbit, Alexa Fluor 594 donkey anti-rabbit and Alexa Fluor 594 donkey anti-goat (all at 1:1000, from Life Tech).

### Quantification of neurogenesis in brain sections

SVZ cells positive for BrdU, DCX, GFAP, NeuN, and nestin were estimated as previously described^29,49^. Positive cells were counted throughout the entire perilesional area or lateral and laterodorsal walls of the lateral ventricles (where the SVZ NPC are located) in every fifth section; 14–16 sections per brain where analyzed under fluorescence microscopy at ×20 magnification. A confocal microscope (OLYMPUS FV1000) was used to take images from triple-labeled brain sections. Mice were coded depending on the treatment and quantification of cells in brain slices was done in blinded analysis.

### Statistical analysis

The data and statistical analysis comply with the recommendations on experimental design and analysis in pharmacology^50^. Statistical analysis was performed using the computer program IBM SPSS Statistics 22. Normal distribution of the data was first analyzed using a Shapiro–Wilks test. Then, a Brown Forsythe test was performed to test the equality of variances. Afterwards, when more than one treatment group were compared, statistical analyses were performed using one-way ANOVA followed by a post-hoc Bonferroni’s test unless otherwise indicated. A Student’s *t* test was used when only one treatment group was compared with the control. Differences were considered significant at values of *p* < 0.05. In general, sample size used in statistical analysis were *n* = 6 for in vivo experiments and *n* = 5–9 for in vitro experiments. Sample sizes were chosen based on a previous works related to this one^8,22,29,47^.

## Supplementary information

Supplementary Figure legends

Supplementary Figure S1

Supplementary Figure S2

Supplementary Figure S3

Supplementary Figure S4

Supplementary Figure S5

Supplmentary Figure S6

Supplementary Figure S7

Supplementary Figure S8

Supplementary table T1

Supplementary movie 1

Supplementary movie 2

Supplementary movie 3

Supplementary movie 4

Supplementary movie 5

Supplementary movie 6

Supplementary movie 7
