# Supplementary material for: A novel PKC activating molecule promotes neuroblast differentiation and delivery of newborn neurons in brain injuries
Source: Cell Death Dis. 2020 Apr 22;11(4):262. doi: 10.1038/s41419-020-2453-9 (PMC7176668; doi:10.1038/s41419-020-2453-9)
Supplement: Supplementary file 1 — Supplementary Figure legends [file 41419_2020_2453_MOESM1_ESM.docx]

**Supplementary Figure S1: Effect of EOF2 on neural progenitor cell (NPC) proliferation *in vitro.*** Proliferation was tested in presence of the basic fibroblastic growth factor (bFGF). **A.** Representative phase-contrast microscopy images of neurospheres cultured for 72 h with or without EOF2 (5µM). Scale bar indicates 200 µm. **B.** Graph shows the effect of EOF2 on neurospheres number expressed as the percentage of control. Data shown are the mean ± S.E.M. of 9 independent measurements. Statistical analysis: * p = 0.02 in two tailed unpaired Student’s t-test comparing EOF2 with the control. **C.** Graph shows the effect of EOF2 on neurospheres area expressed as the percentage of the control. Data shown are the mean ± S.E.M. of 9 independent measurements. **D.** Graph showing PKC activity that was measured in lysates obtained from SVZ-derived NPC that had been treated for 1 hour with EOF2 (5 µM) or none (diluent). Data are the means ± S.E.M; n = 6 independent measurements. Statistical analysis: * p = 0.040 in two tailed unpaired Student’s t-test comparing EOF2 with none.

**Supplementary Figure S2: RNA expression of Neuregulin and ErbB in brain cortical injuries and in the adjacent SVZ.** Ipsilateral mechanical cortical injuries in the primary motor cortex of mice were performed. 14 days post injury the tissue surrounding the injury was isolated as well as the SVZ. Tissue was homogenized and RNA was isolated. mRNA expression of ErbB4 and neuregulin was analyzed by quantitavive real time PCR. mRNA was measured and normalized to the levels of 18s rRNA. Graph shows fold increase based in ΔΔCT. The dotted line represents sham (not lesioned) animals. **A.** ErbB mRNA expression in the injured cortex and in the adjacent SVZ. **B.** Neuregulin mRNA expression in the injured cortex and in the adjacent SVZ. Graph show the ± S.E.M. of 6 animals (n =6). Statistical analysis: * p = 0.033 in Student’s t test comparing with the contralateral hemisphere.

**Supplementary Figure S3: Effect of the local infusion of EOF2 in the neurogenic response of the SVZ to an injury**. **A** Representative confocal microcopy images of the SVZ of adult mice bearing unilateral cortical lesions, locally-infused with vehicle or EOF2 (5 µM). Cortical lesions, BrdU injections and treatments were performed as described in the legend of Fig. 5. Scale bar= 50µm. Dotted lines indicate lateral ventricle walls. Slides were processed for the immunohistochemical detection the proliferation marker BrdU, the neuroblast marker doublecortin (DCX), the glial marker GFAP and the progenitor marker nestin. **B.** Graph represents the average percentage ratios obtained when dividing the number of BrdU^+^ cells in ipsilateral SVZs by the number of BrdU^+^ cells in the corresponding contralateral SVZs (red dashed line). Data are the means ± S.E.M; of six animals n = 6. Statistical analysis: * p = 0.040 in two tailed unpaired Student’s t-test comparing

ipsilateral with contralateral SVZs in a two tailed Student’s t test for paired samples. **C.** Graph shows the number of proliferating cells marked with BrdU per mm^3^ in the ipsilateral SVZ of the indicated animal groups.  **D.** Percentage of BrdU^+^ cells that co-express DCX in the SVZ. **E.** Percentage of BrdU^+^ cells that co-express GFAP in the SVZ. **F.** Percentage of BrdU^+^ cells that co-express nestin in the SVZ. In C, D, and E, data shown are the mean ± S.E.M.; n = 6 animals per group. Statistical analysis: ANOVA and Bonferroni posttest shows no differences.

**Supplementary Figure S4: Effect of the local infusion of EOF2 in the neurogenic response of the DG to an injury**. **A** Representative confocal microcopy images of the DG of the hippocampus of adult mice bearing unilateral cortical lesions, locally-infused with vehicle or EOF2 (5 µM). Cortical lesions, BrdU injections and treatments were performed as described in the legend of Fig. 5. Scale bar= 50µm. Dotted lines indicate the limits of the DG. Slides were processed for the immunohistochemical detection the proliferation marker BrdU, the neuroblast marker doublecortin (DCX), the glial marker GFAP and the progenitor marker nestin. **B.** Graph shows the number of proliferating cells marked with BrdU per mm^3^ in the DG of the indicated animal groups.  **C.** Graph shows the number of neuroblasts DCX^+^ per mm^3^ in the DG of the indicated animal groups. **D.** Percentage of BrdU^+^ cells that co-express DCX in the indicated DG. **E.** Percentage of BrdU^+^ cells that co-express GFAP in the SVZ. **F.** Percentage of BrdU^+^ cells that co-express nestin in the DG. Data shown are the mean ± S.E.M.; n= 6 animals per group. Statistical analysis: 1 Way-ANOVA and Bonferroni posttest shows no differences.

**Supplementary Figure S5: Effect of the intranasal administration of EOF2 in the neurogenic response of the SVZ to an injury**. **A** Representative confocal microcopy images of the SVZ of adult mice bearing unilateral cortical lesions and administrated with vehicle or EOF2 (5 µM) or vehicle. Cortical lesions, BrdU injections and treatments were performed as described in the legend of Fig. 7. Scale bar= 100µm. Dotted lines indicate lateral ventricle walls. Slides were processed for the immunohistochemical detection the proliferation marker BrdU. **B.** Graph shows the number of proliferating cells marked with BrdU per mm^3^ in the ipsilateral and contralateral SVZ of the indicated animal groups. Data shown are the mean ± S.E.M.; n = 6 animals per group. Statistical analysis: * p = 0.0069 in two tailed unpaired Student’s t-test comparing EOF2 ipsilateral vs. contralateral site. * p = 0.1761 in two tailed unpaired Student’s t-test comparing control (vehicle treated mice) ipsilateral vs. contralateral site. * p = 0.0321 in two tailed unpaired Student’s t-test comparing EOF2 ipsilateral vs. Control ipsilateral site

**Supplementary Figure S6: EOF2-induced neuroblast migration from the SVZ towards the injury. A.** Photomerge of representative confocal microscopy images of complete ipsilateral brain sections of adult mice after bearing unilateral cortical lesions and the intranasal administration of EOF2 or only vehicle. Sections were processed for the immune detection of the neuroblast marker doublecortin (DCX). The dotted line indicates the limit of the lesion (L) and the scale bar represent 100 µm in the low magnification pictures and 50 µm in the high magnification picture. **B**. Explanatory drawing of neuroblast migration in EOF2 or only vehicle administrated animals. Neuroblast described a track from the SVZ to the lesioned area in EOF2 administrated animals while in vehicle administrated animals neuroblast only arrived to the CC. Arrows in the figure indicate one the migration pathway to the perilesional area through the CC. Abbreviations: SVZ: subventricular zone; CC: corpus callosum; CTX: cortex.

**Supplementary Figure S7: Effect of the intranasal administration of EOF2 in the phenotypic type of mature neurons in the the perilesional area. A-B.** Representative confocal microcopy images of the SVZ of adult mice bearing unilateral cortical lesions and administrated with EOF2 (5 µM) for 28 days. No BrdU⁺ cells were found within the perilesional area of vehicle treated mice. Cortical lesions and BrdU injections were performed as described in Fig. 7. Scale bar= 50µm. Dotted lines indicate the limits of the lesion. Slides were processed for the immunohistochemical detection of the proliferation marker BrdU, the cholinergic neuron marker ChAT (**A**) and the GABAergic neuron marker Parvalbumin (Parv) (**B**). **C.** Graph shows the number of BrdU cells per mm³ after treatment. Data shown are the mean ± S.E.M.; n = 6 animals per group. Statistical analysis: * p=0.0447 in two tailed unpaired Student’s t-test comparing EOF2 vs. Control. **D.** Graph shows the percentage of BrdU⁺ cells that co-express ChAT or Parv. Data shown are the mean ± S.E.M.; n = 6 animals per group. Statistical analysis: * p=0.0162 in two tailed unpaired Student’s t-test comparing EOF2 vs. Control.

**Supplementary Figure S8: Structural comparisons of EOF2 and ELAC.** (A) Conformation of EOF2, consistent with selected nuclear Overhauser enhancements (red arrows) observed by NOESY2D experiments; for an equivalent conformation of ELAC (see reference 21). (B) Superimposed conformations of ELAC (R_1_=H, R_2_=H, R_3_=Tg) and EOF2 (R_1_=COCH_2_(*p*-MeO)Ph, R_2_=R_3_=Ac); atoms and functional groups in red are considered to be involved in hydrogen-bond interactions with PKC. Abbreviations: Ac, acetyl; Tg, tigloyl.

| **mRNA target of HEK293T cells** | **Average Ct** | **Primers used** | **Amplicon size** | **Relative abundance** |
| --- | --- | --- | --- | --- |
| **Classical PKCs** |  |  |  |  |
| PKC α | 27,34 | Fw:CAAGGTTCATGCAGCCCAAC | 322 | +++ |
|  |  | Rw:ACTGTGTCCCTGGCAAAACA |  |  |
| PKC β | 33,53 | Fw:GACCAAACACCCAGGCAAAC | 182 | + |
|  |  | Rw:GATGGCGGGTGAAAAATCGG |  |  |
| PKC γ | NA | Fw:GAGATCCCGCCTCCTTTCAG | 229 | - |
|  |  | Rw:CTGGGGTGCAGGATATGACG |  |  |
| **Novel PKCs** |  |  |  |  |
| PKC ε | 28,32 | Fw:CGGCGAGGAAATACATGCAC | 147 | +++ |
|  |  | Rw:GGGCAGGAATGAAGAACCGA |  |  |
| PKC η | 35,74 | Fw:GGTGCTGAAGAAGGACGTGA | 140 | + |
|  |  | Rw:AAAACAGACGATCGGGGGTC |  |  |
| PKC δ | 36,29 | Fw:TGGTTGGTGCGTTGTAGCAG | 119 | + |
|  |  | Rw:TAGGAGTTGAAGGCGATGCG |  |  |
| PKC θ | 31,50 | Fw:GAAACCTCAAGGCCGAATGC | 173 | ++ |
|  |  | AGAAGGTGGCAGTGAACTCG |  |  |
| **Atypical PKCs** |  |  |  |  |
| PKC λ | 27,76 | Fw:GATGAGGAAGGAGACCCGTG | 237 | +++ |
|  |  | Rv:CCTGTTGAAACGCTTGGCTT |  |  |
| PKC ζ | 29,15 | Fw:GACATGTGTCGTCTGCACCAG | 162 | ++ |
|  |  | Rw:GGTGCTCGGGAAAACATGAA |  |  |
| **Metalloprotease** |  |  |  |  |
| ADAM10 | 33,98 | Fw:GGGGGCTGTGCAGATCATTC | 285 | + |
|  |  | Rw:GATTCCGGAGAAGTCTGTGGT |  |  |
| ADAM17 | 35,71 | Fw:GGGAAAAGAGGATTGAGGGGC | 291 | + |
|  |  | Rw:TCTTACCGAATGCTGCTGGA |  |  |
|  |  |  |  |  |
|  |  |  |  |  |
| **Supplementary table 1:** Measurement of the mRNA expression of different PKCs and metalloproteases in HEK293T cell line. Total RNA was isolated from HEK293T cultures and subjected to reverse transcription and real-time qPCR. The mRNAs were measured. The table shows mRNA abundance in relation to the most-abundant PKC α mRNA. The table also shows average Ct values (PCR-cycle number in which the fluorescence of the amplified sequence becomes detectable) for each targeted mRNA. | | | | |
|  |  |  |  |  |
|  |  |  |  |  |
|  |  |  |  |  |
|  |  |  |  |  |
|  |  |  |  |  |
