# Supplementary figures and images for: A novel PKC activating molecule promotes neuroblast differentiation and delivery of newborn neurons in brain injuries
Source: Cell Death Dis. 2020 Apr 22;11(4):262. doi: 10.1038/s41419-020-2453-9 (PMC7176668; doi:10.1038/s41419-020-2453-9)

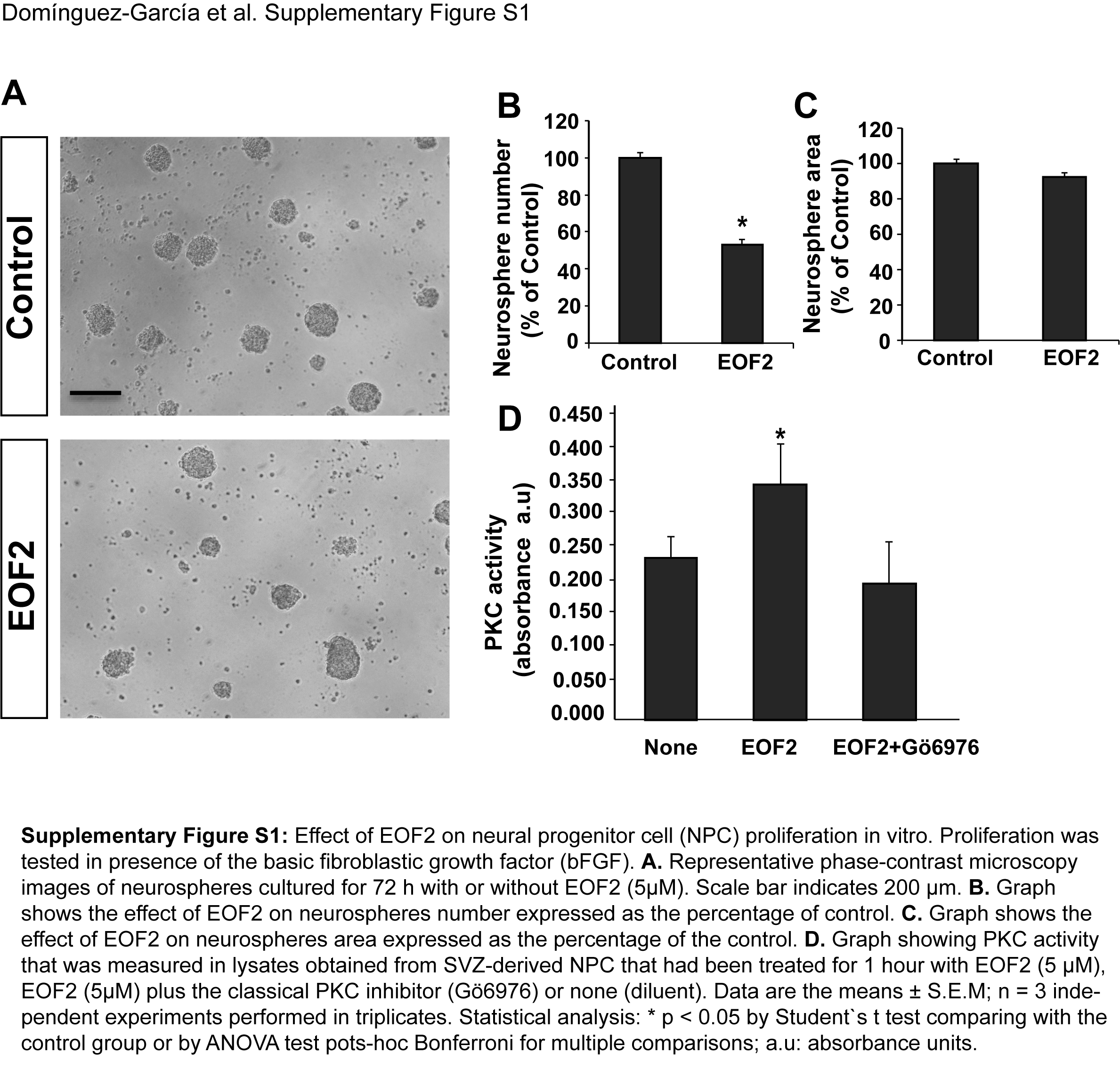

Supplement: Supplementary file 2 — Supplementary Figure S1 [file 41419_2020_2453_MOESM2_ESM.tif]

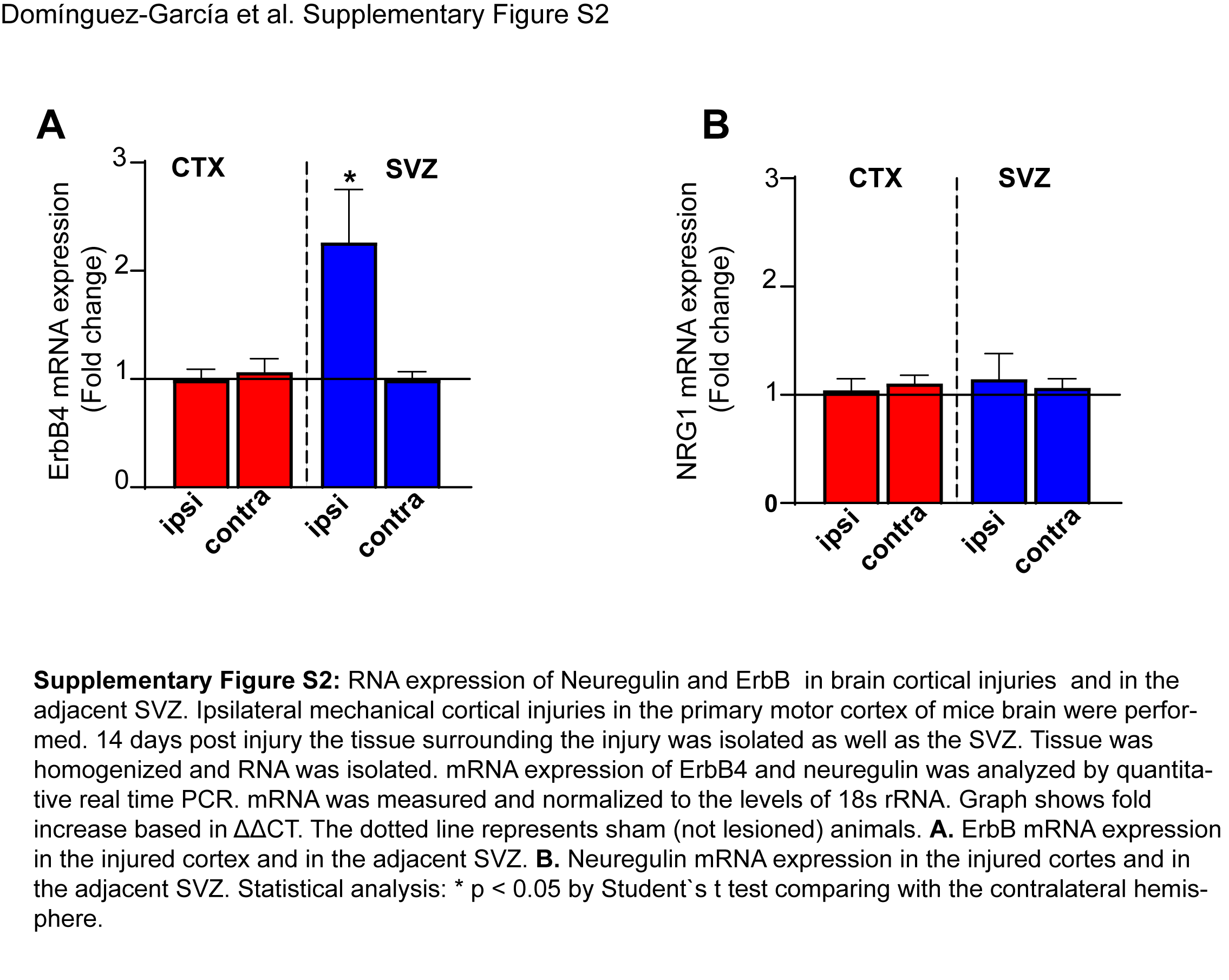

Supplement: Supplementary file 3 — Supplementary Figure S2 [file 41419_2020_2453_MOESM3_ESM.tif]

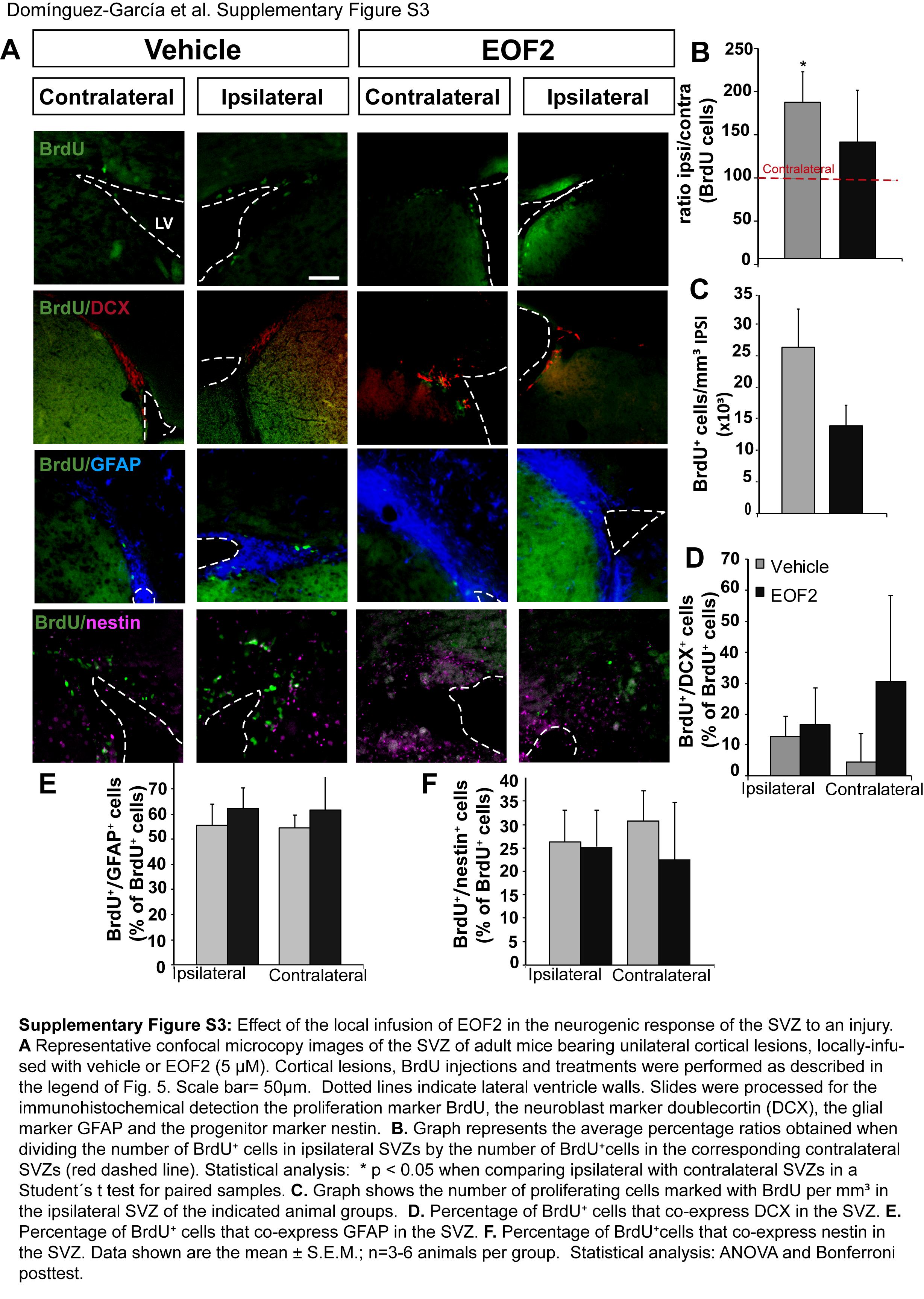

Supplement: Supplementary file 4 — Supplementary Figure S3 [file 41419_2020_2453_MOESM4_ESM.tif]

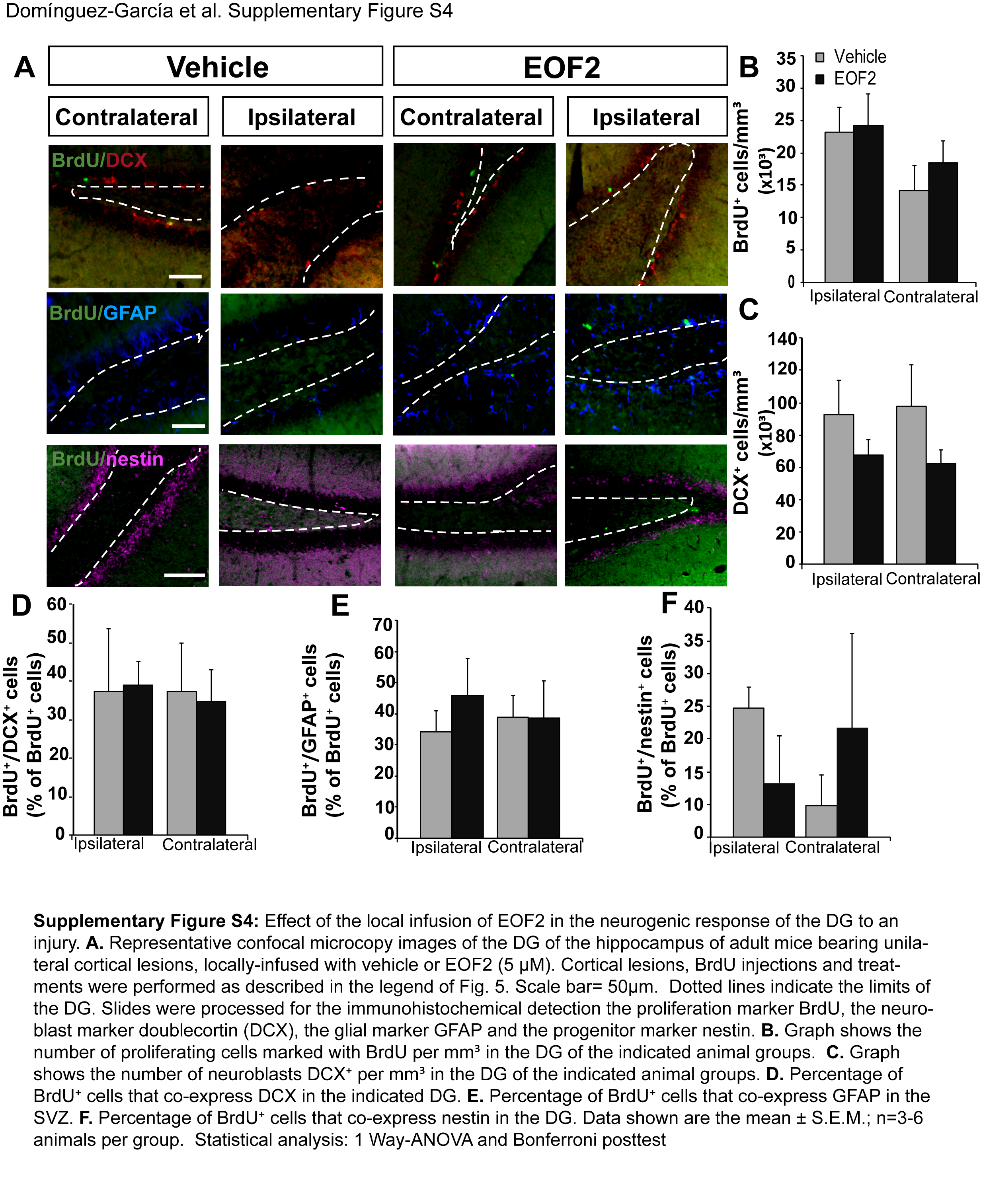

Supplement: Supplementary file 5 — Supplementary Figure S4 [file 41419_2020_2453_MOESM5_ESM.tif]

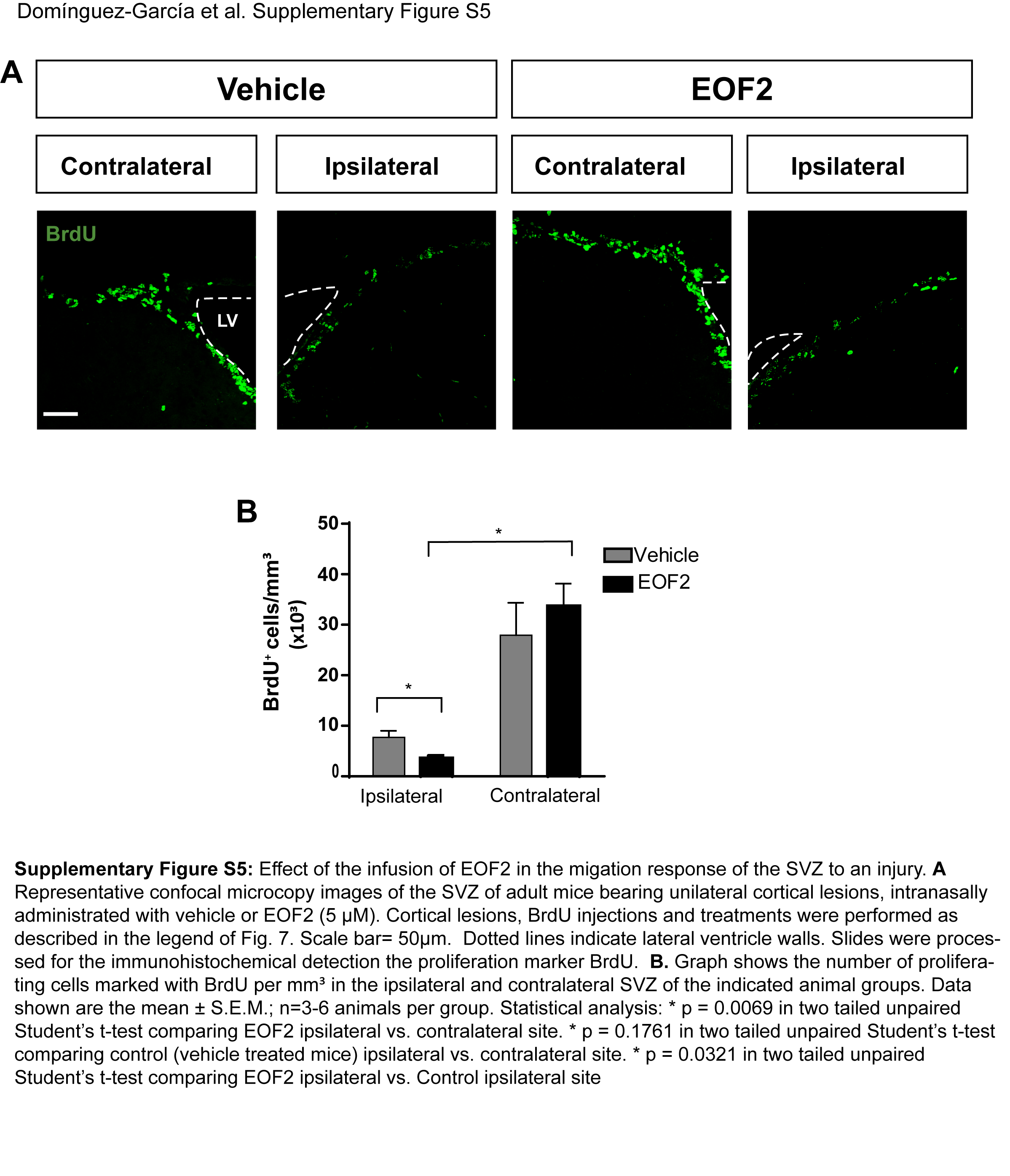

Supplement: Supplementary file 6 — Supplementary Figure S5 [file 41419_2020_2453_MOESM6_ESM.tif]

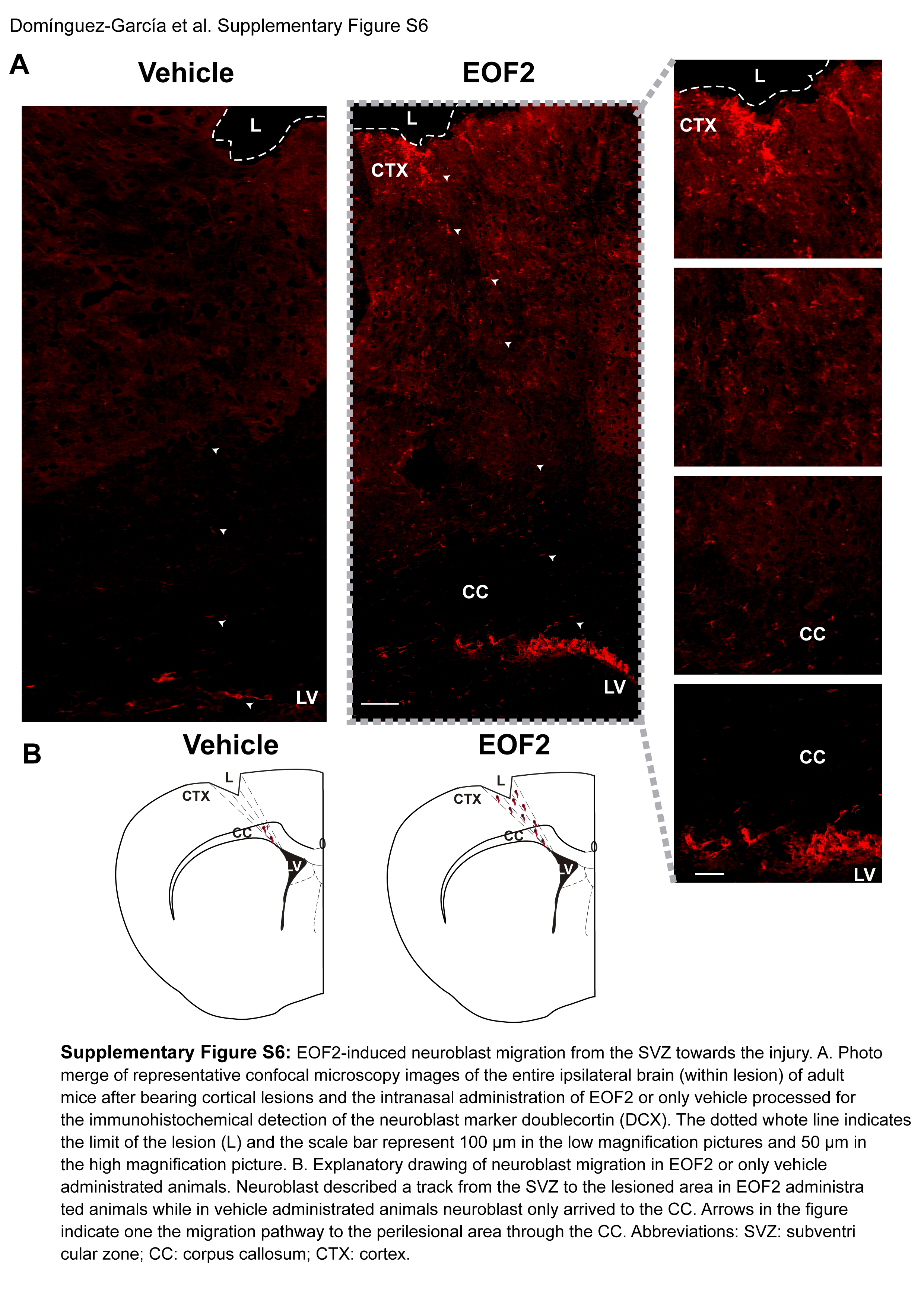

Supplement: Supplementary file 7 — Supplmentary Figure S6 [file 41419_2020_2453_MOESM7_ESM.tif]

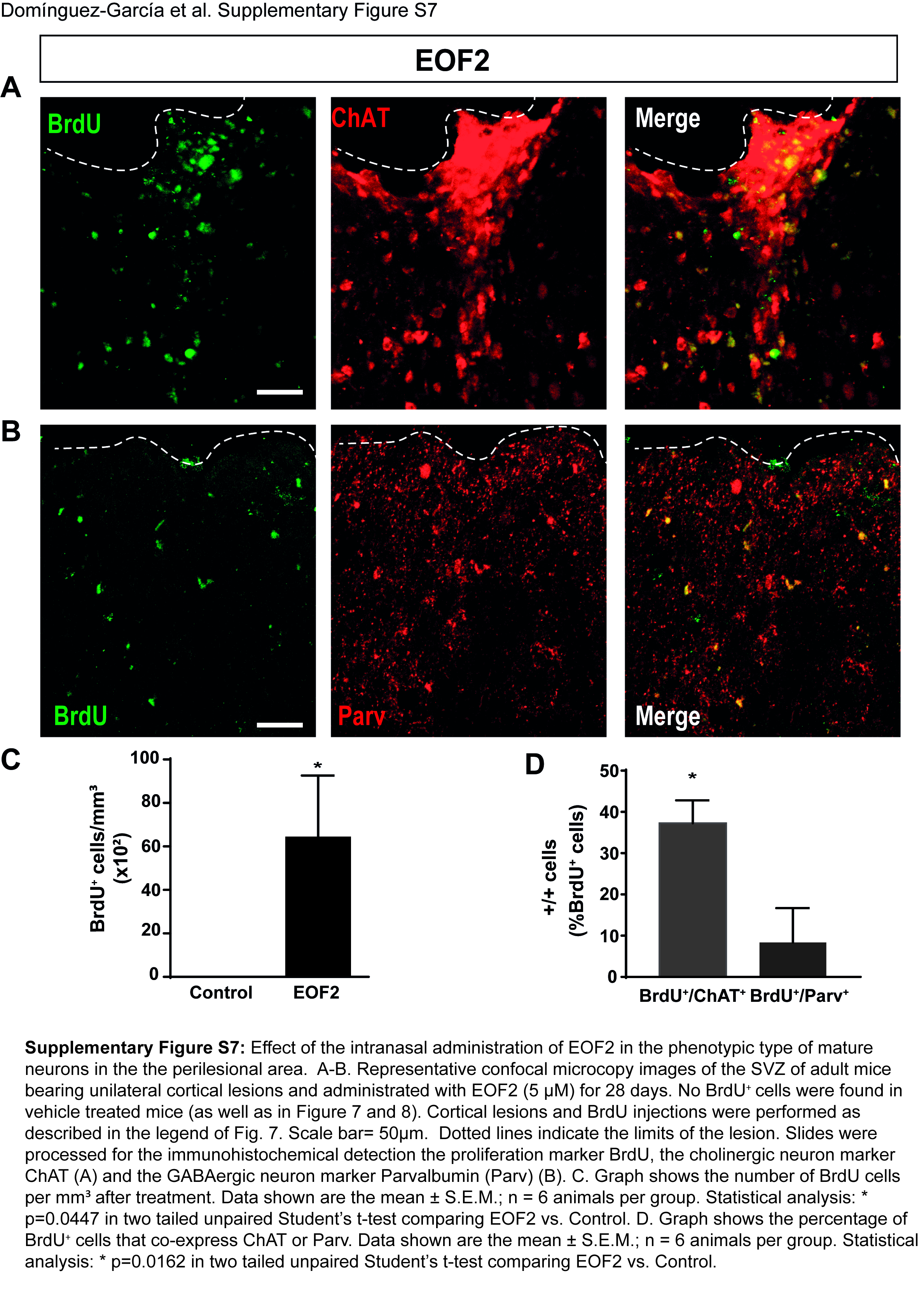

Supplement: Supplementary file 8 — Supplementary Figure S7 [file 41419_2020_2453_MOESM8_ESM.tif]

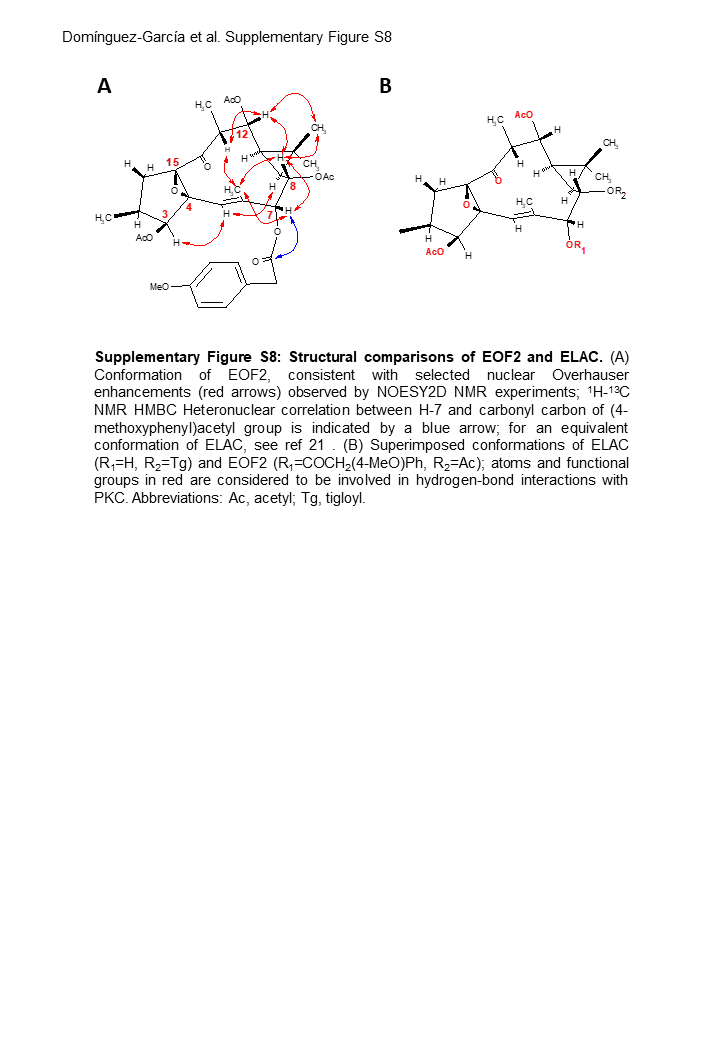

Supplement: Supplementary file 9 — Supplementary Figure S8 [file 41419_2020_2453_MOESM9_ESM.tif]
