# Supplementary material for: A novel PKC activating molecule promotes neuroblast differentiation and delivery of newborn neurons in brain injuries
Source: Cell Death Dis. 2020 Apr 22;11(4):262. doi: 10.1038/s41419-020-2453-9 (PMC7176668; doi:10.1038/s41419-020-2453-9)
Supplement: Supplementary file 10 — Supplementary table T1 [file 41419_2020_2453_MOESM10_ESM.docx]

| **mRNA target of HEK293T cells** | **Average Ct** | **Primers used** | **Amplicon size** | **Relative abundance** |
| --- | --- | --- | --- | --- |
| **Classical PKCs** |  |  |  |  |
| PKC α | 27,34 | Fw:CAAGGTTCATGCAGCCCAAC | 322 | +++ |
|  |  | Rw:ACTGTGTCCCTGGCAAAACA |  |  |
| PKC β | 33,53 | Fw:GACCAAACACCCAGGCAAAC | 182 | + |
|  |  | Rw:GATGGCGGGTGAAAAATCGG |  |  |
| PKC γ | NA | Fw:GAGATCCCGCCTCCTTTCAG | 229 | - |
|  |  | Rw:CTGGGGTGCAGGATATGACG |  |  |
| **Novel PKCs** |  |  |  |  |
| PKC ε | 28,32 | Fw:CGGCGAGGAAATACATGCAC | 147 | +++ |
|  |  | Rw:GGGCAGGAATGAAGAACCGA |  |  |
| PKC η | 35,74 | Fw:GGTGCTGAAGAAGGACGTGA | 140 | + |
|  |  | Rw:AAAACAGACGATCGGGGGTC |  |  |
| PKC δ | 36,29 | Fw:TGGTTGGTGCGTTGTAGCAG | 119 | + |
|  |  | Rw:TAGGAGTTGAAGGCGATGCG |  |  |
| PKC θ | 31,50 | Fw:GAAACCTCAAGGCCGAATGC | 173 | ++ |
|  |  | AGAAGGTGGCAGTGAACTCG |  |  |
| **Atypical PKCs** |  |  |  |  |
| PKC λ | 27,76 | Fw:GATGAGGAAGGAGACCCGTG | 237 | +++ |
|  |  | Rv:CCTGTTGAAACGCTTGGCTT |  |  |
| PKC ζ | 29,15 | Fw:GACATGTGTCGTCTGCACCAG | 162 | ++ |
|  |  | Rw:GGTGCTCGGGAAAACATGAA |  |  |
| **Metalloprotease** |  |  |  |  |
| ADAM10 | 33,98 | Fw:GGGGGCTGTGCAGATCATTC | 285 | + |
|  |  | Rw:GATTCCGGAGAAGTCTGTGGT |  |  |
| ADAM17 | 35,71 | Fw:GGGAAAAGAGGATTGAGGGGC | 291 | + |
|  |  | Rw:TCTTACCGAATGCTGCTGGA |  |  |
|  |  |  |  |  |
|  |  |  |  |  |
| **Supplementary table 1:** Measurement of the mRNA expression of different PKCs and metalloproteases in HEK293T cell line. Total RNA was isolated from HEK293T cultures and subjected to reverse transcription and real-time qPCR. The mRNAs were measured. The table shows mRNA abundance in relation to the most-abundant PKC α mRNA. The table also shows average Ct values (PCR-cycle number in which the fluorescence of the amplified sequence becomes detectable) for each targeted mRNA. | | | | |
|  |  |  |  |  |
|  |  |  |  |  |
|  |  |  |  |  |
|  |  |  |  |  |
|  |  |  |  |  |
